# Refinement of learned skilled movement representation in motor cortex deep output layer

**DOI:** 10.1038/ncomms15834

**Published:** 2017-06-09

**Authors:** Qian Li, Ho Ko, Zhong-Ming Qian, Leo Y. C. Yan, Danny C. W. Chan, Gordon Arbuthnott, Ya Ke, Wing-Ho Yung

**Affiliations:** 1 School of Biomedical Sciences, Faculty of Medicine, The Chinese University of Hong Kong, Hong Kong, China; 2 Department of Medicine and Therapeutics, Faculty of Medicine, The Chinese University of Hong Kong, Hong Kong, China; 3 Li Ka Shing Institute of Health Sciences, Faculty of Medicine, The Chinese University of Hong Kong, Hong Kong, China; 4 Gerald Choa Neuroscience Centre, Faculty of Medicine, The Chinese University of Hong Kong, Hong Kong, China; 5 Chow Yuk Ho Technology Center for Innovative Medicine, Faculty of Medicine, The Chinese University of Hong Kong, Hong Kong, China; 6 Laboratory of Neuropharmacology, School of Pharmacy, Fudan University, Shanghai 201203, China; 7 Okinawa Institute of Science and Technology Graduate University, Okinawa, Japan 904-0495

## Abstract

The mechanisms underlying the emergence of learned motor skill representation in primary motor cortex (M1) are not well understood. Specifically, how motor representation in the deep output layer 5b (L5b) is shaped by motor learning remains virtually unknown. In rats undergoing motor skill training, we detect a subpopulation of task-recruited L5b neurons that not only become more movement-encoding, but their activities are also more structured and temporally aligned to motor execution with a timescale of refinement in tens-of-milliseconds. Field potentials evoked at L5b *in vivo* exhibit persistent long-term potentiation (LTP) that parallels motor performance. Intracortical dopamine denervation impairs motor learning, and disrupts the LTP profile as well as the emergent neurodynamical properties of task-recruited L5b neurons. Thus, dopamine-dependent recruitment of L5b neuronal ensembles via synaptic reorganization may allow the motor cortex to generate more temporally structured, movement-encoding output signal from M1 to downstream circuitry that drives increased uniformity and precision of movement during motor learning.

## Abstract (web summary)

Motor learning induces structural and functional reorganization in upper layers of motor cortex. Here the authors show that neuronal ensembles in the output layer 5b exhibit temporal dynamics during skilled learning that progressively becomes well-aligned to movement in a dopamine dependent manner.

The capability to learn novel, complex motor skills is a remarkable ability of human and many other mammals. Although multiple extracortical brain areas, notably the cerebellum and the basal ganglia, contribute to motor learning^1^, it has been argued that the primary motor cortex (M1) is ideally suited not only for the execution of movement but also the acquisition and storage of motor memory^2^. Recent finding also highlights the essential role of the motor cortex in tutoring subcortical motor circuits in acquiring motor skills^3^. M1 neurons are known to comprise a functionally heterogeneous population, encoding distinct motor parameters such as direction, velocity, position and muscle activity^4^,^5^,^6^,^7^,^8^,^9^,^10^, while jointly they may constitute a pattern generator to encode for the large diversity of possible movements^11^. The motor representations of individual neurons and neuronal ensembles in M1 are not static, exhibiting adaptation to task requirements, as well as improved coding and predictability of behavioural outcomes with motor learning^12^,^13^,^14^,^15^,^16^,^17^.

M1 circuitry exhibits interlaminar specificity. Layer 2/3 (L2/3) provides excitatory input to layer 5a (L5a) and layer 5b (L5b), while L5a also relays feedforward excitatory drive to L5b (refs 18, 19, 20, 21). Functional imaging in animals undergoing motor training demonstrated that M1 L2/3 neurons consist of functionally distinct types with different sensory, motor and decision correlates during motor task which are modulated by learning^22^,^23^. In the forelimb area of M1, after repeated lever-manipulation task L2/3 neurons exhibit more reproducible population activity in relation to motor execution^24^. Interestingly, L2/3 neurons retain similar predictability of motor outcome during training of lever-press task, while L5a neurons become progressively recruited for the task and a substantial proportion of neurons become more predictive of lever trajectory after motor training^25^. However, it remains unknown, how these learning induced reorganizations of neuronal coding in L2/3 and L5a are eventually conveyed to and reflected in the motor representation of the output layer L5b, which is beyond the reach of optical functional imaging techniques (for example, two-photon microscopy) due to its depth. Previous studies showed that M1 neurons encode movement parameters with temporal leads or lags on the order of tens to hundreds of milliseconds^5^,^8^,^9^,^26^,^27^; however, how these temporal dynamics in motor representation in M1 change with learning remains virtually unknown.

It has been suggested that reorganization of both the interlaminar and intrinsic horizontal connections in M1 underlies learning-induced plasticity of motor representation. Great insight has been provided by recent *in vivo* imaging studies of the motor cortex, which revealed rapid changes in spine dynamics on the apical dendrites of L5 neurons within the first few hours^28^,^29^,^30^. These findings suggest a crucial role of long lasting synapse remodelling and connectivity reorganization in the formation as well as consolidation of motor memory. Electrophysiological studies, on the other hand, have revealed that motor skill training could strengthen horizontal connections in the superficial layers 1–3 of the motor cortex, manifested as an increase in local field potentials (FPs) evoked in cortical slices studied *in vitro*^31^,^32^. However, up to now, physiologically induced long-term potentiation (LTP) during motor skill learning has not been demonstrated convincingly and tracked *in vivo* in the motor cortex.

To probe the process of motor learning and the refinement of motor representation by L5b, we performed a longitudinal study by chronic recording of single-unit activities from M1 in rats performing repetitive motor skill task up to 7 days. We elucidated the fine-scale temporal dynamics of single neuron and population activities from ensembles of L5b neurons, as well as the maintenance of training-induced synaptic plasticity *in vivo*. We also interrogated the process by examining the effect of depleting dopamine, the neuromodulator that has been consistently implicated in different types of cortical plasticity and learning^33^,^34^,^35^.

## Results

### *In vivo* recordings in L5b during forelimb-reaching training

**Figure 1:**
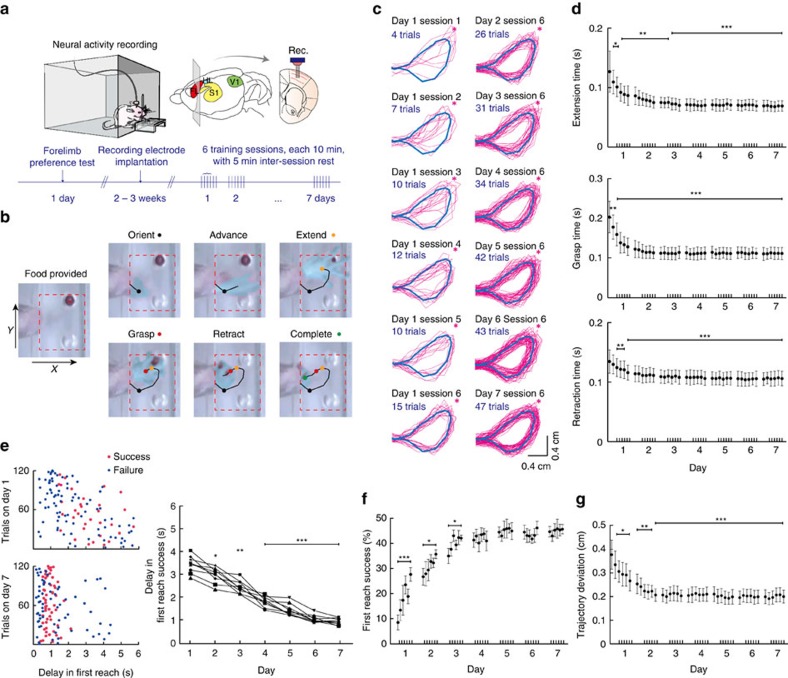
Forelimb reaching for food training and simultaneous recording from L5b neurons in M1. (**a**) Schematics of experimental paradigm. Neural activities in L5b were recorded during forelimb food-reaching task training over 7 days by multi-channel recording electrode array (Rec.). FL and HL: forelimb and hindlimb territories of M1; S1: primary somatosensory cortex; V1: primary visual cortex. (**b**) Six phases of a first reach success trial captured by camera and the forelimb trajectory tracked automatically (see Methods). (**c**) The evolution of more uniform forelimb trajectories (pink) in first reach success trials. The reference expert trajectory is shown in blue colour. Red asterisk denotes the position of food pellet. (**d**) The duration of forelimb extension (upper panel), grasping (middle panel) and retraction (lower panel) in first reach success trials. The timing of reaching action shortened significantly in day 1 and exhibited further decrease in day 2 and 3, and remained steady thereafter. Mean±s.d. *P<0.05; **P<0.01; ***P<0.001, one-way repeated measures ANOVA, *n*=9. (**e**) Left, delay in first reach attempt quantified by the time interval between food provision and the ‘orient' position of forelimb on days 1 and 7 (120 consecutive trials each from a single representative rat). Right, learning associated shortening in the delay in first reach success attempt. Mean±s.d. of delay in first reach success trials: day 1: 3.44±0.37 s; day 7: 0.96±0.14 s, *P*=2.51 × 10^−6^; *P<0.05; **P<0.01; ***P<0.001, all compared with day 1; one-way ANOVA, 9 rats; first reach failure trials: day 1: 2.31±1.55 s; day 7: 1.45±1.20 s, *P*=0.012; one-way ANOVA, 9 rats. (**f**) Training-dependent improvement in first reach success rate (see text). (**g**) Evaluation of forelimb trajectory spatial variance as the averaged distance integrated over time between the actual trajectories in first reach success trials (pink) and the reference expert trajectory (blue) shown in **d**. Mean cumulative Euclidean distance±s.d., day 1 session 1: 0.375±0.061 cm; day 1 session 6: 0.265±0.042 cm, *P=*0.0173; day 2 session 1: 0.252±0.041 cm, *P*=0.0052; day 2 session 6: 0.205±0.026 cm, *P=*7.85 × 10^−4^; *P<0.05; **P<0.01; ***P<0.001, all compared to day 1 session 1, one-way repeated measures ANOVA, *n*=9.

To investigate the temporal dynamics of single-neuron and population activities in L5b during motor learning, food-restricted rats (*n*=9) were trained with a forelimb reaching and grasping task for 7 consecutive days (Fig. 1a, Supplementary Movies 1 and 2, left panel), with simultaneous multi-channel single-unit recordings at L5b of the forelimb territory in M1 (Supplementary Fig. 1a). To acquire the largest possible samples of neurons per animal, we employed 16-channel microwire array for recording from neuronal ensembles. Data were included for analyses only if correct targeting of electrodes at L5b was verified by post-mortem histological reconstruction of the recording sites, based on that L5b is distinct from neighbouring L5a and layer 6 (L6) with a denser VGlut2 immunoreactivity and larger neuronal soma size^36^,^37^,^38^ (Supplementary Fig. 1a,b; see Supplementary Fig. 1c for all reconstructed recording sites from one animal). Furthermore, to provide enough single-unit data from the same animal for neuronal population analyses, we only included rats from which more than 20 single-units were recorded in L5b (see Supplementary Fig. 1d,e for the locations of all the recording sites included for analyses).

Each animal received six 10-min training sessions per day, with 5-min rest intervals between sessions. The reaching attempts involved a coordinated motor sequence consisting of different phases: orienting, advancing, extending, grasping, retracting and completion (Fig. 1b, see Methods for definition of each phase), resulting in trackable forepaw trajectories (Fig. 1c). Over days of training, the extension time, grasp time and retraction time of individual first reach success trials (defined as trials during which the animal completed the reaching attempt and consumed the food pellet successfully on the first reaching attempt) exhibited progressive decrease and became less variable from trial to trial, especially over the first 2 days of training and then became steady (Fig. 1d). In addition, rats responded to the provision of food with progressively shorter and less variable delays in first reach success trials (Fig. 1e). We also observed the largest increase in the proportion of first reach success trials during the first 3 days, both within and across days, which levelled off from day 4 (Fig. 1f, mean±s.e.m. of first reach success rate: day 1 session 1: 8.7±3.0% versus session 6: 28.2±2.8%, *P*=2.03 × 10^−4^; day 2 session 1: 27.3±3.4% versus session 6: 36.4±1.8%, *P*=0.020; day 3 session 1: 35.7±3.1% versus session 6: 42.9±1.2%, *P*=0.037; day 4 session 1: 42.1±2.9% versus session 6: 41.8±4.0%, *P*=0.913; day 7 session 1: 45.6±2.1% versus session 6: 46.6±1.1%, *P*=0.659; one-way repeated measures ANOVA, 9 rats). Notably, the acquired motor skill memory was retained overnight. Improved skillfulness was also reflected in increased spatial uniformity of forepaw movement trajectory, especially during first reach success trials in the first 2 days of training, as both the average and variance of deviation from the reference expert trajectory (computed from the average of 50 randomly selected first success trials from day 7 session 6, see Fig. 1c and Methods) decreased significantly (Fig. 1g).

### Long-term stability of chronic single-unit recordings in L5b

**Figure 2:**
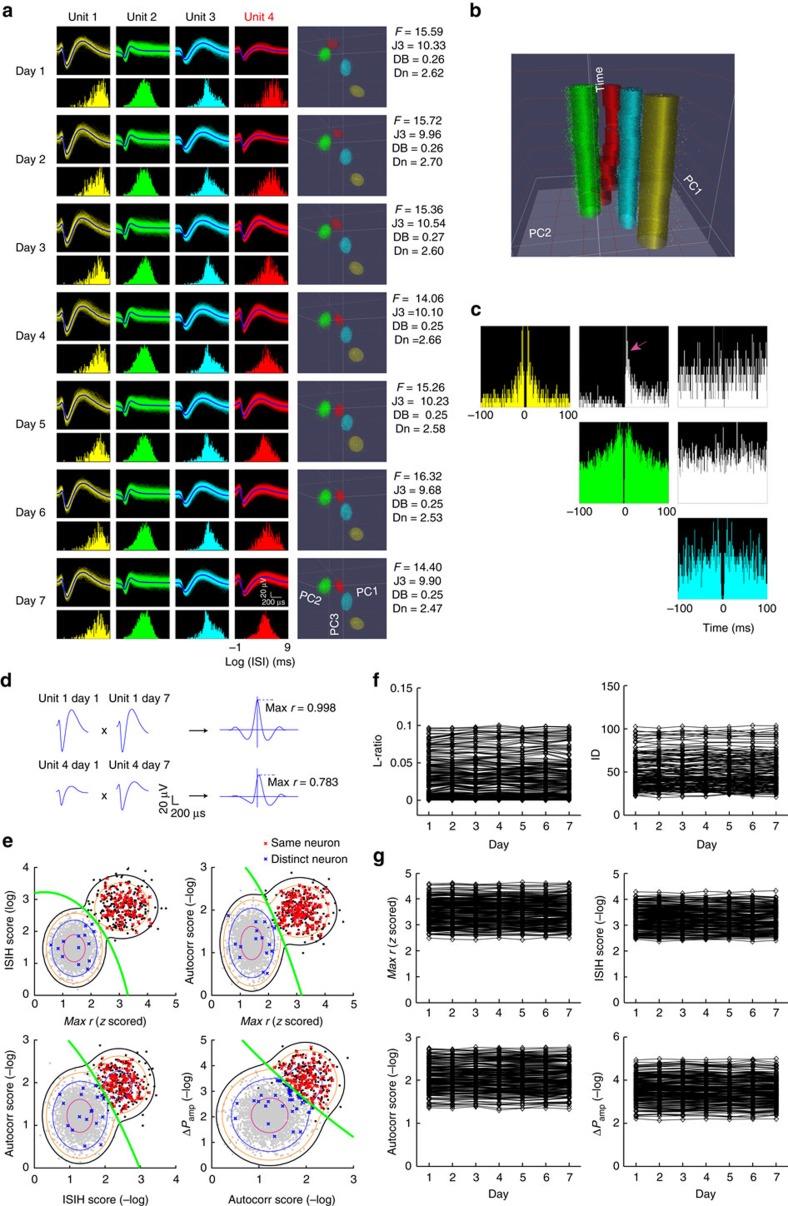
Spike sorting and assessment of long-term stability of single-unit recordings by single microwire array over 7 days. (**a**) Example of spike sorting from single microwire array in 7 days, showing the superimposed spike waveforms (upper panel) and the inter-spike-interval histogram (ISIH, lower panel), and the corresponding identified clusters in the PCs space (far right panel). Clear isolation of units from a given recording channel is indicated by high, F statistic of MANOVA (F), J3, Dunn validity (Dn) and low Davis-Bouldin (DB) index (see Methods). Note the excluded unit in red, whose spike waveforms changed cross days, and had shifted ISI histogram and cluster location in PCs. (**b**) Long-term stability of identified single-units shown in **a** over 7 days. The unit shown in red with drifting of cluster was excluded. (**c**) Autocorrelograms of the three isolated units and their cross-correlogram (white). The presence of refractory periods in the auto-correlograms and absence of refractoriness in the cross-correlogram indicated spikes with clusters marked in yellow, green and blue were generated by three distinct neurons. The short latency sharp peak in the cross-correlogram (arrow) between the putative pyramidal neuron (yellow, reference of the cross-correlogram) and the interneuron (green) may indicate mono-synaptic activation. (**d**) Example of units exhibiting stable (top, unit 1 in **a**) or unstable (bottom, unit 4 in **a**) spike waveform are shown in **d**. (**e**) Gaussian mixture distributions fitted to combinations of the four similarity scores (see Methods) computed from spikes recorded from same neuron (black dots, representing true positive values computed using recording acquired in difference sessions on the same day, see Methods) or distinct ones (grey dots, computed from recordings from different channels simultaneously) on the same day corresponding contours: 50% (red), 95% (blue), 99.9% (orange), 99.97% (black) of the distribution. Red and blues crosses represented recordings classified as stably corresponding to the same or arising from distinct neurons respectively, based on combining the use of multiple similarity scores with quadratic classifiers (green lines). (**f**) Cross-day stability of single-unit isolation quality assessed by L-ratio and isolation distance (*n*=158 included units shown in red in **e**). (**g**) Cross-day stability of four cluster similarity scores (*n*=158 included units shown in red in **e**, day 1 session 1 recordings were used as reference).

To study the properties of motor skill representation during the 7-day training process, it was critical that only well-separated units that exhibited long-term stability were included for further analyses. We validated the stability of single-unit tracking by comparison against standard tetrode recordings. The quality of single-unit isolation was assessed by computing quantitative measures of cluster quality, from both 16-channel microwire array and tetrodes. Specifically, the criteria for single-unit are: signal-to-noise ratio>4, isolation distance (ID)^39^ ≥ 15, L-ratio≤0.2 (refs 40, 41, 42, 43), and a clear refractory period revealed by both inter-spike interval (ISI) distribution and auto-correlograms with 99.5% of events with ISI>2 ms (microwire array: Fig. 2a–c, Supplementary Fig. 2a–c; tetrode: Supplementary Fig. 2d–f, see Methods).

To determine whether a unit was stable and represented correct tracking of the same neuron over consecutive days, we computed four criterion scores: maximum time-shifted linear correlation coefficient of spike waveform (*Max r*, Fig. 2d), normalized spike peak-to-peak amplitude difference (Δ*P*_amp_), dissimilarity score for inter-spike interval histogram (ISIH), and dissimilarity score for autocorrelation histogram^44^,^45^,^46^,^47^ (see Methods). We fitted Gaussian mixture model to distributions of combinations of the criteria and employed quadratic discriminant analysis to obtain optimal decision boundary for classification of recordings that correspond to the same or distinct neuron (Fig. 2e, Supplementary Fig. 2g,h; see Methods for details). We chose the *Max r*–ISIH dissimilarity score joint distribution as the optimal discrimination model because these were the two most informative features that gave the lowest Bayesian information criterion (BIC) and Akaike information criterion (AIC) (see Methods). We classified 158 single units as stable tracking of the same cells through 7 days (see Supplementary Fig. 2i for estimated cumulative false positive and negative rates; also see Methods). 47 units from microwire array recording and 16 units from tetrode recordings disappeared into background or occurred in transit thus failed to be tracked over 7 days. Sorting quality (microwire array, Fig. 2f; tetrodes, Supplementary Fig. 2j) and tracking stability (microwire array, Fig. 2g; tetrodes, Supplementary Fig. 2k) for all included single-units remained stable throughout the 7 training days. We therefore concluded that, in our experiments, the single-unit tracking performance of microwire array recordings was comparable to that of tetrode recordings.

### Diversity in single neuron dynamics in L5b during learning

**Figure 3:**
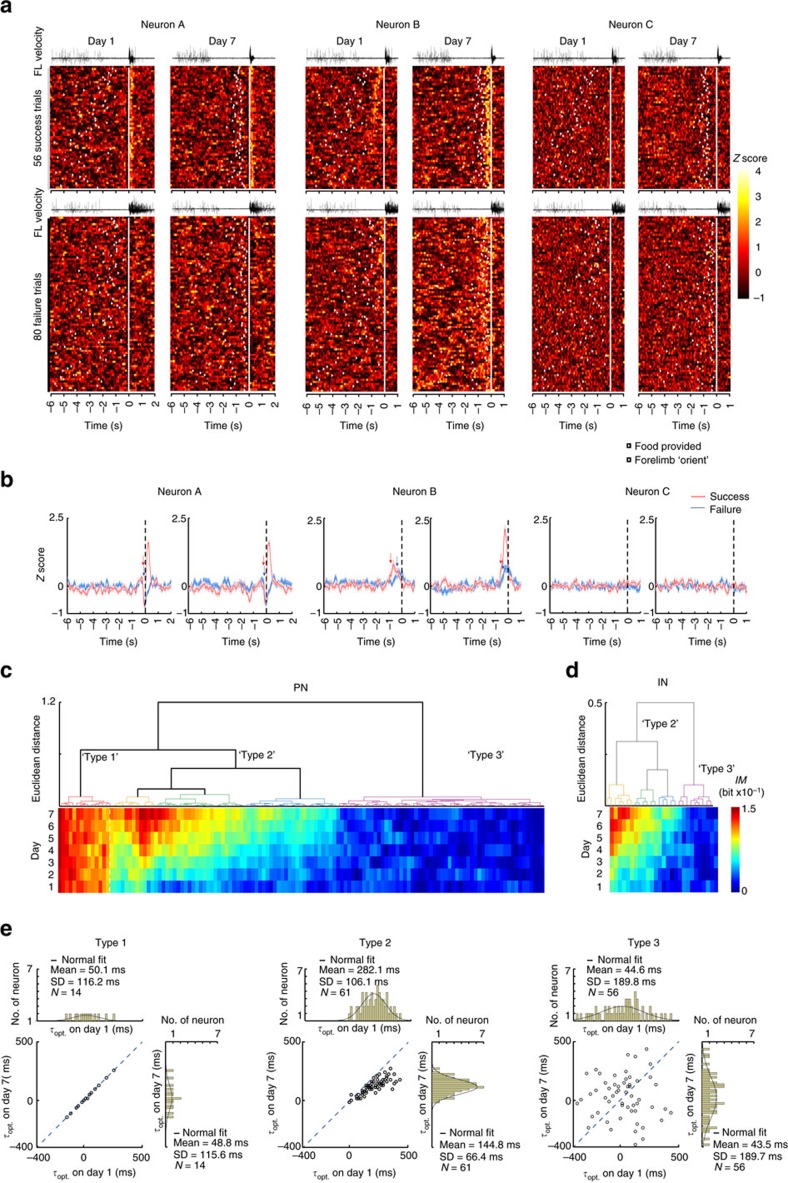
Fine-temporal scale refinement of firing-motor output relationship in a subpopulation of L5b PNs. (**a**) Three examples of L5b PNs' peri-event time histograms, aligned by the ‘orient' position (time ‘0', white dots). Fifty-six consecutive first reach success and 80 consecutive first reach failure trials are stacked. White arrows indicate the time when food pellets were provided. The correspondent forelimb velocity, are shown overlaid on the top. Neural activities were normalized and expressed as Z score. Neuron A's firing highly correlated with forelimb action but remained unchanged with training. Neuron B's firing became correlated with forelimb action after training. Neuron C's firing did not correlate with forelimb reaching action irrespective of training. (**b**) Averaged neural activities of the three L5b PNs shown in **a** during first reach success (red, mean±s.e.m.) and first reach failure (blue, mean±s.e.m.) trials on days 1 and 7. Arrows indicate the time when the neural activity began to diverge (TUD, time until divergence, see Methods). (**c**) Hierarchical clustering of 131 L5b PNs (recorded from five rats) based on single neuron *I*_*M*_ during motor learning. The dendrogram (upper half) depicts Euclidean distance of single PN *I*_*M*_ vectors across 7 training days, with major subgroups indicated by different colours in the dendrogram. (**d**) Twenty-seven L5b INs (recorded from five rats) were classified into subgroups by applying the same method of hierarchical clustering of single neuron *I*_*M*_ as shown in **c**. (**e**) Summary of training-dependent changes of the optimal time lag of *I*_*M*_ (*τ*_opt._ for the three major types of L5b PNs (recorded from five rats) classified by hierarchical clustering. Frequency histograms of individual neuron *τ*_opt._ at days 1 and 7 are shown on the top and the right respectively. Sixty out of sixty-one of type 2 neurons exhibit a decrease in *τ*_opt._, which are distributed below the dashed line with unit slope.

The 158 L5b neurons, collected from 61 channels on microwire arrays from five out of nine rats included for analyses, were classified putatively as either pyramidal neurons (PNs, 131/158) or interneurons (INs, 27/158) based on their electrophysiological properties (Supplementary Fig. 3a–c). On the first day of experiments, we invariably observed a spectrum of activities of these neurons, with some clearly showing firing correlated with forelimb displacement and velocity and others that were less correlated. Analysis of single neuron dynamics revealed that 10.7% (14/131) of PNs exhibited reaching-correlated firing as shown in their peri-event time histograms (PETH) (Fig. 3a,b, neuron A) which were temporally aligned to the ‘orient' position (see Methods and Fig. 1b). This reaching-correlated firing property did not change with training (see Supplementary Fig. 4a,b for further examples). On the other hand, 44.3% (58/131) PNs' activities initially had no or little correlation with reaching execution, but with learning, their activities also became temporally aligned to forelimb movement with either increase (37/58, Fig. 3a,b, neuron B, also see Supplementary Fig. 4c,d for further examples) or decrease in firing (21/58, Supplementary Fig. 4e,f for further examples). There were also 45.0% (59/131) of PNs whose peri-event firing changes did not reach statistical significance despite training (Fig. 3a,b, neuron C). These results suggest that the firing characteristics of a subpopulation of L5b neurons are not static, but progressively change with motor learning.

### Fine-temporal scale refinement of skilled movement encoding

Previous studies demonstrated increased information content of M1 neuronal firing about motor output in monkeys^15^ as well as M1 L5a neurons in rodents^25^ with motor learning, while the time scale at which firing–motor output relationship may be altered remains elusive. Having observed L5b PNs with diverse behavioural correlates, we next investigated the modulation of peri-task execution firing and information content by motor learning in these individual L5b PNs, as well as the time scale of the changes. We computed the mutual information between single-unit instantaneous firing rate and forelimb instantaneous velocity as a function of different time lags (*τ*, ranging from −500 to +500 ms, with *τ*>0 meaning firing precedes instantaneous movement, Supplementary Fig. 5a), and obtained the optimal time lag (*τ*_opt._) for each neuron, defined as the value of *τ* at which the mutual information attains maximum (*I*_*M*_).

Hierarchical clustering of single neuron over the 7 days (Fig. 3c for PNs; Fig. 3d for INs) and analyses of the associated changes in *τ*_opt._ (Fig. 3e) confirmed sub-groups of PNs that responded differently to training. Of all 131 PNs analysed, one subgroup (named Type 1 neurons, including neuron A in Fig. 3a,b, 14/131 or 10.7% of PNs from five rats) showed robust *I*_*M*_ regardless of the day of training (Fig. 3c, Type 1, see Supplementary Fig. 5b for neurons from one representative rat) and had constant *τ*_opt._ (Fig. 3e, left panel). For these type 1 neurons, a rapid increase in *I*_*M*_ and relatively constant *τ*_opt._ was already apparent on the first training day (Supplementary Fig. 5c, neuron A and ex. 2; Supplementary Fig. 5d,e, Type 1). Thus, they carried robust information about forelimb movement velocity right from the beginning of, and throughout motor training. In contrast, another subgroup of PNs (named Type 2 neurons, including neuron B in Fig. 3a,b, 61/131 or 46.6% of PNs from five rats) exhibited progressive increase in *I*_*M*_ of different degrees (Fig. 3c, Type 2; see Supplementary Fig. 5b for neurons from one rat), and interestingly, was always associated with a reduction in *τ*_opt._ after the first training day (59/61 of type 2 PNs exhibited reduction in *τ*_opt._ on day 7 versus day 1; mean change of *τ*_opt._±s.d.=−84.2±70.5 ms, or 37.3% reduction, *P<*10^−5^, paired *t*-test, Fig. 3e, middle panel). During the first training day, increase in *I*_*M*_ for Type 2 neurons was already evident and *τ*_opt._ emerged from random to exhibiting consistent values (Supplementary Fig. 5c, neuron B, Supplementary Fig. 5d,e, Type 2). The remaining PNs (named Type 3 neurons, including neuron C in Fig. 3a,b, 56/131 or 42.7% of PNs from five rats) had insignificant *I*_*M*_ (Fig. 3c, Type 3; see Supplementary Fig. 5b for neurons from one rat) and scattered *τ*_opt._ throughout training (Fig. 3e, right panel). Consistent with these observations, analysis of single neuron activity revealed that, after training, type 2 PNs exhibited earlier changes in PETH, demonstrated by a shortening in time until divergence (mean±s.d. of time until divergence, day 1 session 6: 265.12±85.58 ms, day 7 session 6: 173.13±71.98 ms, *P<*10^−5^, paired *t*-test, Supplementary Fig. 5f, see Methods). Statistical significance of single neuron *I*_*M*_ over 7 days was estimated by bootstrap resampling (Supplementary Fig. 5g, see Methods).

To account for the confounding factor of reduction of motor execution time causing possible apparent shortening of *τ*_opt._, we performed additional analyses whereby the variability of duration of the attempts was eliminated by mapping the forepaw trajectory of individual attempts to the reference expert trajectory by dynamic time warping (DTW), which finds the optimal mapping for each individual forelimb trajectories to the reference trajectory. The mapping found was then applied to the time series data of neural activities and the forelimb instantaneous velocity, and *τ*_opt_ was then re-calculated. After controlling for the variation in timing of individual reaching attempts, type 1 neurons still exhibited consistent *τ*_opt._ (Supplementary Fig. 5h), whereas a shortening of *τ*_opt._ was still observed specifically for type 2 neurons (Supplementary Fig. 5i).

### L5b neurons predict motor outcome with increasing accuracy

**Figure 4:**
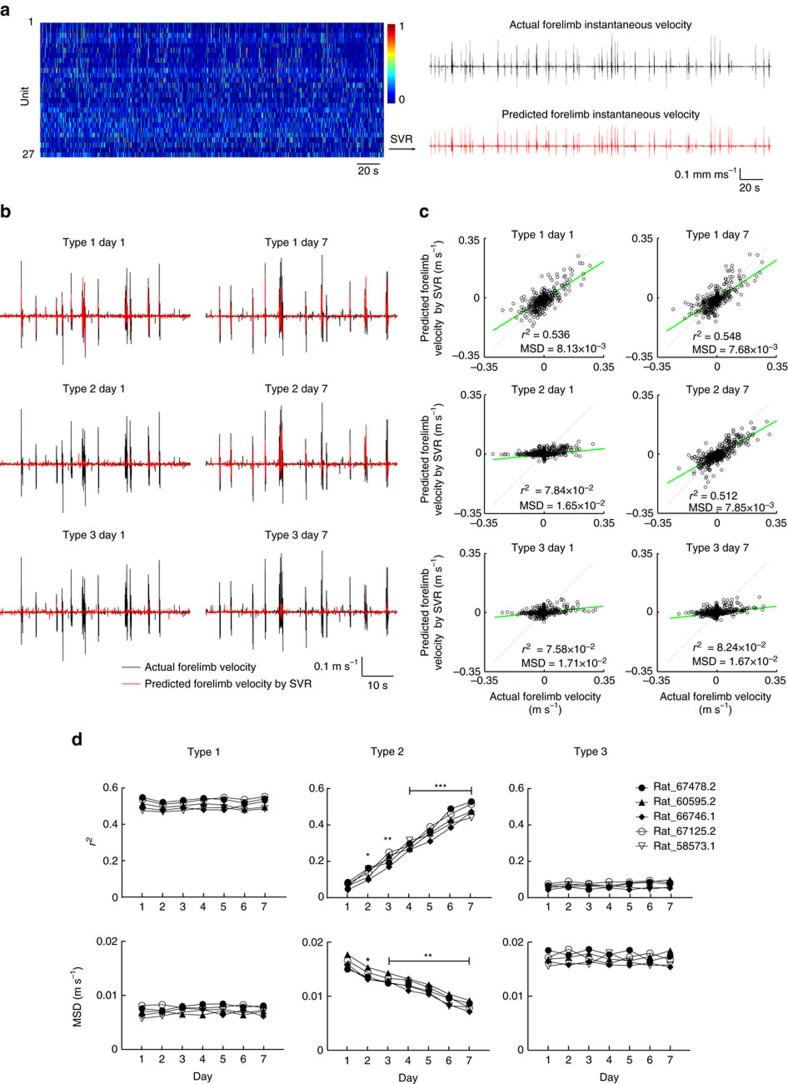
Learning-dependent changes of population prediction accuracy for forelimb instantaneous velocity. (**a**) SVR decoding of forelimb velocity from neural population activities. For each neuron, the firing histogram (bin size: 12.5 ms, left) was aligned to the behavioural event (27 units from an example animal), and the values was linearly normalized to 0–1 range. The actual forelimb instantaneous velocity (top right) was predicted using SVR by the corresponding population spike events. (**b**) Representative traces of actual forelimb instantaneous velocity (black) and the SVR model predicted forelimb velocity (red) by three types of neurons classified (type 1 neuron: *n*=4; type 2 neuron: *n*=12; type 3 neuron: *n*=11, from an example animal), illustrating the changes in population decoding accuracy during early (day 1) and late (day 7) training sessions. (**c**) Least squares regression analyses between actual forelimb instantaneous velocity and the SVR model predicted forelimb velocity based on three types of L5b PNs shown in **a** during early and late training sessions. The Pearson's correlation coefficient (*r*^2^) and mean squared deviation (MSD) for each regression are shown. Each data point represents the instantaneous velocity of the forelimb trajectory predicted from neural population activity versus the actual velocity of displacement calculated from high-speed camera recording (in 12.5 ms bins). (**d**) Summarized result of *r*^2^ and MSD of predicted and actual forelimb instantaneous velocity by three types of PNs (*n*=131) recorded from five rats. Upper left panel, day 1: *r*^2^=0.513±0.013, day 3: *r*^2^=0.505±0.010, *P*=0.098; day 7: *r*^2^=0.520±0.013, *P*=0.492; bottom left panel, day 1: MSD=0.0069±0.00043, day 3: MSD=0.0073±0.00028, *P*=0.370; day 7: MSD=0.0072±0.00033, *P*=0.448. Top middle panel, day 1: *r*^2^=0.067±0.007, day 3: *r*^2^=0.211±0.014, *P*=0.006; day 7: *r*^2^=0.483±0.016, *P*=1.23 × 10^−4^; Bottom middle panel, day 1: MSD=0.0161±0.00048, day 3: SD=0.0130±0.00034, *P*=0.008; day 7: MSD=0.0081±0.00035, *P*=0.002. Top right panel, day 1: *r*^2^=0.061±0.006, day 3: *r*^2^=0.066±0.015, *P*=0.587; day 7: *r*^2^=0.073±0.008, *P*=0.312. Bottom right panel, day 1: MSD=0.0169±0.00047, day 3: MSD=0.0171±0.00051, *P*=0.282; day 7: MSD=0.0167±0.00055, *P*=0.781, all compared to day 1, one-way repeated measures ANOVA, *n*=5.

Given an increase in mutual information between single-unit firing and motor parameters among PNs recruited for the task, we then determined how well motor behaviour variables could be decoded from type 2 neuronal ensemble activities in comparison to type 1 neurons, and if decoding performance from different neuronal subpopulations changes with learning. We employed support vector regression (SVR) to perform time series forecasting of forelimb instantaneous velocity (Fig. 4a) and forelimb displacement (Supplementary Fig. 6a–c) (see Methods). For each neuron, we performed single neuron SVR decoding using activities within a time window preceding velocity and found that the optimal window size that enabled the highest prediction accuracy (*τ*_SVR_) was invariably greater than or equal to *τ*_opt._ (Supplementary Fig. 6d; also see Methods). To maximize prediction accuracy, we used preceding population activities within a time window covered by the maximum *τ*_*SVR*_ among the neuronal population for prediction of instantaneous velocity. Decoding accuracy was quantified by Pearson's correlation coefficient (*r*^2^) and mean squared deviation between the actual and decoded forelimb velocities (Fig. 4b–d) and displacements (Supplementary Fig. 6a–c). Type 1 neurons activity predicted forelimb velocity with the highest fidelity throughout training (Fig. 4d, left panel). Decoding accuracy from type 2 neuron activities, in contrast, was training-dependent, whereby significant and progressive improved decoding of forelimb velocity from these neurons could be obtained throughout the 7 training days (Fig. 4d, middle panel). Consistent with a lack of change in information about motor parameters over the 7 days of motor learning, type 3 neurons remained non-informative about forelimb velocity (Fig. 4d, right panel).

To assay tuning stability of the different types of neurons, we used SVR trained on the data of each day for prediction of movement velocity on the previous day. For type 1 neuron ensembles, SVR trained on each day had similar prediction accuracies for same-day and previous-day velocity prediction (Supplementary Fig. 6e,f, Type 1). Interestingly, for type 2 neuron ensembles, SVR model trained on data from each day had slightly higher prediction accuracies for previous-day velocity than same-day data (Supplementary Fig. 6e,f, Type 2). These results indicate that both type 1 and type 2 neurons exhibited information and tuning stability, and suggest ongoing consolidation of coding by type 2 neurons during motor training.

### Learning induces task-specific correlation structures in L5b

**Figure 5:**
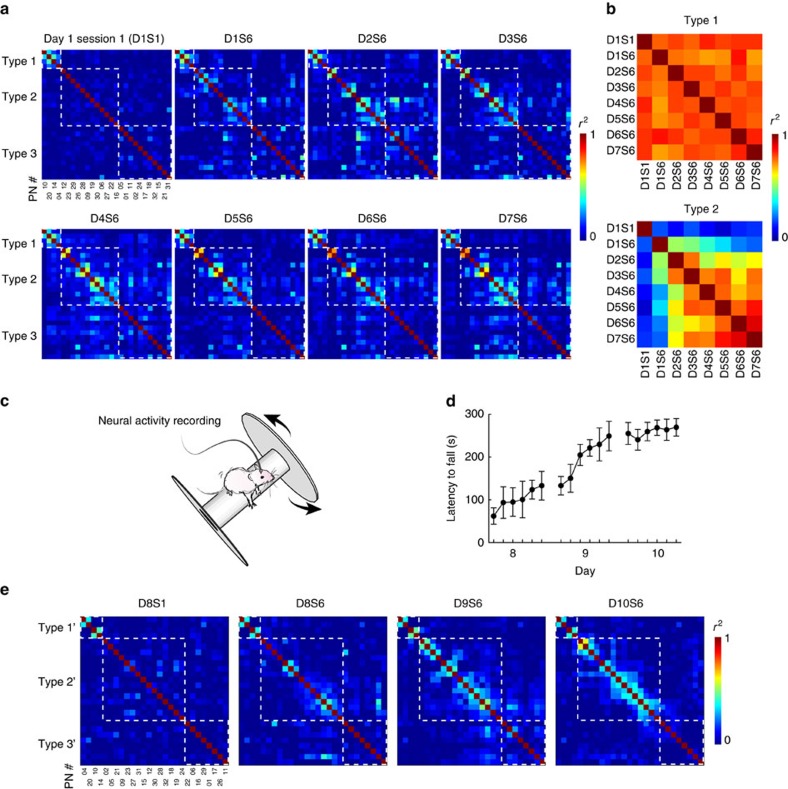
Emergence of correlation structure of L5b PNs during motor learning. (**a**) Pairwise cross-correlation matrix of 27 L5b PNs across 7 training days, recorded from one representative rat during first reach success attempts (controlled for trajectory variance, see Methods). Neurons are ordered (PN #) according to the sequence of hierarchical clustering shown in Supplementary Fig. 5b. The squares from top to bottom segregate type 1 to type 3 L5b PNs identified. Increased correlation is evident only among the groups of neurons that show increased *I*_*M*_ and decrease of *τ*_opt._, that is, type 2 neurons. (**b**) Summary of the preserved overall similarity of cross-day correlation matrix among type1 and type 2 PNs. Each colour-coded element represented the averaged similarity index of cross-day correlation matrices from five rats. (**c**) Paradigm of rotarod running. Rats were trained to run on the rotarod accelerating from 4 to 40 revolutions per minute over 300 s. Each trial ended when the rat fell off or when 300 s was reached. Each animal received six training sessions every day, and each lasted 10 min with 5-min rest intervals. (**d**) Latency to fall off the rotarod during training. Animals showed fast improvement in performance on the first two days and maintained throughout the third day (mean±s.e.m. of latency to fall in day 1 session 1: 62.0±19.1 s, day 1 session 6: 133.0±33.4 s, *P*=0.051; day 2 session 1: 132.1±21.7 s, day 2 session 6: 249.0±34.6 s, *P*=0.007; day 3 session 1: 255.2±25.7 s, day 3 session 6: 269.5±20.3 s, *P*=0.681, all compared to day 1 session 1, one-way repeated measures ANONA, 4 rats). (**e**) Pairwise cross-correlation matrix among the 27 L5b PNs shown in **a**, but re-ordered for clustering with high correlation coefficient near the diagonal during 3 days' rotarod training (day 8 to day 10).

As learning-induced changes in neuronal correlation and population activity pattern in M1 upstream layers that provide excitatory drive to L5b have previously been reported^22^,^24^, we next investigated how joint-neuronal firing statistics in L5b may be shaped by learning. To exclude the possibility that the cross-trial variability of neuronal activities reflects merely the changes in forelimb kinematics, we controlled for the forelimb trajectory variability by selecting first reach success trials with actual trajectories closely approximating the reference expert trajectory from each individual day (criteria: 30 randomly selected first reach success trials with trajectory deviation within mean±s.d. of cumulative Euclidean distance after DTW, see Methods) and restricted the pairwise cross-correlation analysis only to the period from the time of ‘food provided' to ‘complete' state. We observed that type 1 neurons exhibited clear correlated activities on day 1 of training and the correlation structure was robustly maintained throughout the 7 days (mean±s.e.m. of correlation coefficient in day 1 session 1: *r*^2^=0.257±0.051; day 1 session 6: *r*^2^=0.268±0.061, *P*=0.886; day 3 session 6: *r*^2^=0.258±0.067, *P*=0.986; day 7 session 6: *r*^2^=0.263±0.057, *P*=0.632; all compared to day 1 session 1, one-way repeated measures ANOVA, 14 pairs of type 1 neurons from five rats, see Fig. 5a for six pairs of type 1 PNs from one representative rat). In contrast, among type 2 neurons which were originally weakly correlated, clusters of neurons with correlated activities emerged in the first day of motor learning and the correlation structure was further strengthened during later phase of training (mean±s.e.m. of correlation coefficient in day 1 session 1: *r*^*2*^=0.037±0.009; day 1 session 6: *r*^*2*^=0.047±0.007, *P*=0.164; day 3 session 6: *r*^*2*^=0.128±0.018, *P*=0.006; day 7 session 6: *r*^*2*^=0.154±0.016, *P*=8.45 × 10^−4^; all compared to day 1 session 1, one-way repeated measures ANOVA, 348 pairs of type 2 neurons from five rats; see Fig. 5a for 66 pairs of type 2 PNs from one rat). On the other hand, type 3 neurons did not show robust structured activity correlation throughout the training period. Assessment of the overall similarity of correlation matrix across 7 training days of the type 1 and type 2 PNs (Fig. 5b) and INs (Supplementary Fig. 7a,b) from all rats suggested the emergence of task-specific neural engram among these neurons.

We also computed correlations for type 1 and type 2 neurons using spontaneous activities recorded when the animal was not executing the task, and found that only the similarity of correlation matrix for type 2 neurons was slightly strengthened towards the later period of training (type 1: day 1 session 1: *r*^2^=0.045±0.015 versus day 7 session 6: *r*^2^=0.055±0.011, *P*=0.364; 14 pairs of type 1 neurons from five rats; type 2: day 1 session 1: *r*^2^=0.039±0.009; day 7 session 6: *r*^2^=0.067±0.013, *P*=0.037; 348 pairs of type 2 neurons from five rats; see Supplementary Fig. 7c for 6 pairs of type 1 and 66 pairs of type 2 PNs from the same rat as shown in Fig. 5a; see Supplementary Fig. 7d for overall similarity of correlation matrices across 7 days).

To verify the task-specificity of the correlated activities of the type 2 neurons, after 7 days of training, we switched the animal to another motor skill learning task, the rotarod test, and continued the recordings from the same single-units for 3 more days (Fig. 5c). The rotarod test also requires the participation of forelimb muscles but in a different context. Gauged by the latency to fall from the rotarod, the animals showed quick improvement on the first two days (Fig. 5d,e). The pattern of activity correlation emerged during forelimb reaching test was not observed when the animal performed the rotarod test, and a different pattern of correlation was apparent during the test (Fig. 5e, 27 PNs from the same neuronal populations in Fig. 5a, ordered for clustering of high correlation coefficient near the diagonal). Our results suggest that the recruitment of a subpopulation of PNs for a task may involve the selection and strengthening of horizontal recurrent excitatory inputs or shared long range inputs from other areas to L5b PNs that are involved in specific task execution.

### Emergence of reproducible spatiotemporal activities in L5b

Previous data suggest the emergence of more uniform neurodynamics of type 2 neurons during motor training. To elucidate the basal firing activities of type 1 and type 2 neurons and their modulation by motor training, we analysed the spontaneous firing rates of type 1 and type 2 neurons, and quantified neural variance by mean-matched Fano factor (FF)^48^. Type 1 neurons had in general higher spontaneous firing rate than type 2 neurons (Supplementary Fig. 7e, mean firing rate±s.d., type 1: 9.2±3.5 Hz; type 2: 5.9±4.0 Hz, *t*-test, *P=*0.0054), which suggest that type 1 neurons may more likely be corticopeduncular neurons, while type 2 neurons may more likely correspond to corticostriatal neurons^49^. Apart from a steady *I*_*M*_ and *τ*_opt._, for type 1 neurons a significant decrease in firing variance within a time window near forelimb grasping was already evident during day 1 session 1 (Supplementary Fig. 8a, day 1 session 1, mean-matched FF±s.e.m., pre-grasp: 2.189±0.133, during grasp: 1.706±0.114, *P=*0.021; post-grasp: 2.161±0.125, *P=*0.717; all compared to pre-grasp; one-way ANOVA, *n*=14). This relationship strengthened rapidly within the first training day and persisted throughout the training period (Supplementary Fig. 8a, day 1 session 6 and day 7 session 6). For type 2 neurons, emergence of significant reduction of mean-matched FF occurred within the first day of motor training and persisted throughout the training period (Supplementary Fig. 8b).

**Figure 6:**
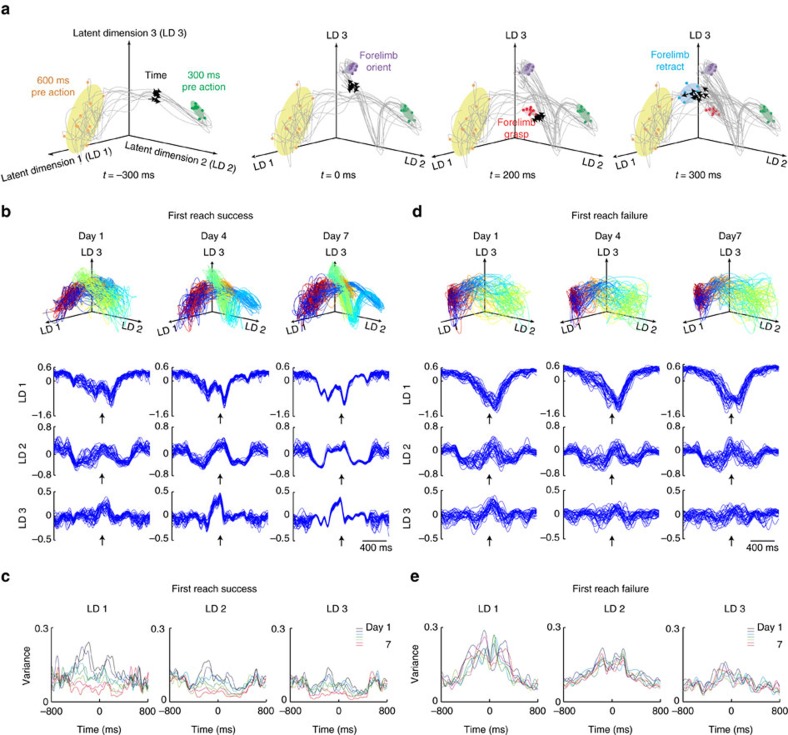
Illustration of single-trial neural trajectories extracted from population neural activities. (**a**) The temporals of ten single-trial neural trajectories extracted from first reach success trials by applying GPFA (see Methods) embedded in the top three orthonormalized latent dimensional space (LD1-3) spanning from 800 ms before to 800 ms after the ‘orient' position, derived from 27 L5b PNs recorded from an representative animal. The black arrows indicate the flow of time series and coloured dots denote different stages of forelimb action. The ellipses indicate the across-trial variability (two s.d. around the mean) at these states. (**b**) and (**d**) Top: Neural trajectories of 30 randomly selected first reach success trials with trajectory deviation within mean±s.d. of cumulative Euclidean distance from reference expert trial after DTW, and neural trajectories of 30 randomly selected first reach failure trials, derived from 27 L5b PNs' activities recorded from the same example animal. The flow of time series is colour gradient coded, from blue (start) to red (end). Bottom: the same neural trajectories shown in three individual latent dimensional space (LD1-3). Arrow indicates ‘orient' position. (**c**) and (**e**) Analysis of the variance of the neural trajectories in LD1-3, quantified by the diagonal matrix of the covariance ellipsoids, showing progressive reduction in the variance in first reach success trials (**c**) but not in failure trials (**e**) during training.

To further probe whether motor learning is associated with the emergence of reproducible population activity of L5b neurons during task execution, we applied the Gaussian-process factor analysis algorithm (GPFA)^50^, a dimensionality reduction algorithm, to extract smooth single-trial neural trajectories from PN population activities during individual reaching attempts (Fig. 6a). The neural trajectories were embedded in a three-dimensional space composed by the top three-orthonormalized latent dimensions, which together accounted for 89.4±3.9% (mean±s.d., five rats) of the total variance. To control for the potential contribution of increased uniformity of forelimb trajectory to any changes in neural variance observed, we selected first reach success trials with forelimb trajectories closely approximating the reference expert trajectory from each individual day (criteria: 30 randomly selected first reach success trials with trajectory deviation within mean±s.d. of cumulative Euclidean distance after DTW, see Methods) and extracted neural population activity trajectories from these trials. As training progressed, the neural trajectories progressively exhibited reduction in variance (see Methods) during first reach success trials over the entire 7-day training period (Fig. 6b,c, data from one typical rat, and Supplementary Fig. 9a–d, data from two more rats), despite that the variance of task execution time and forelimb trajectories as well as proportion of first reach success trials reached steady levels (see Fig. 1e,g), but not during first reach failure trials (Fig. 6d,e, 30 randomly selected first reach failure trials on each day). We also computed neural trajectories separately for type 1 and type 2 neurons, and observed that the decreased variance of neural trajectories with training could be attributed to type 2 neurons only (Supplementary Fig. 9e,f, Type 1; Supplementary Fig. 9g,h, Type 2). Therefore, a reduction in neural trajectory variance was not a mere reflection of reduced forelimb trajectory variance, but represents reorganization of L5b population PNs neural activities with motor learning especially during successful execution of desirable movement, a phenomenon largely attributable to more reproducible activities of type 2 neurons.

### Physiologically induced synaptic plasticity in L5b

**Figure 7:**
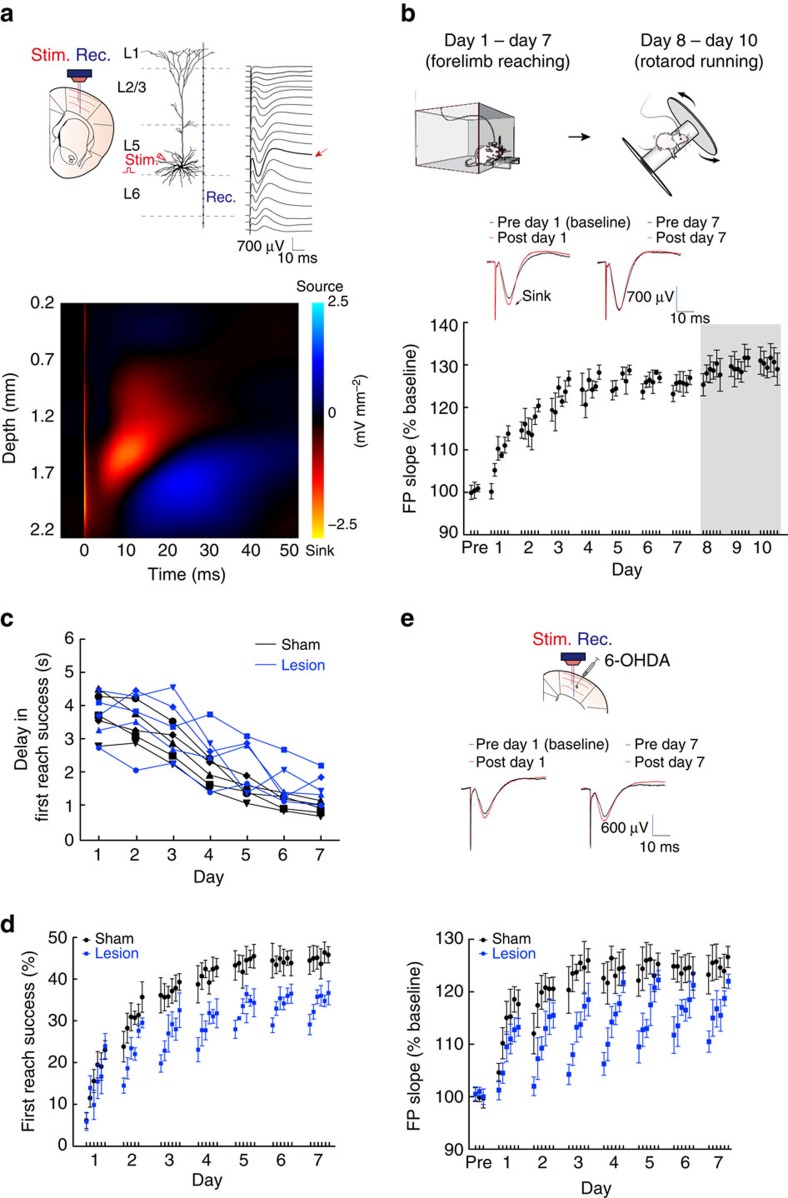
Local dopamine depletion impaired training-induced LTP of synaptic inputs. (**a**) Top, averaged traces of field potentials (FPs) evoked *in vivo* at multiple sites from L1 to L6 of M1, recorded via 20 recording contacts in a linear microprobe (Rec.). The stimulating electrode (Stim.) was placed at L5, which could activate synaptic inputs to the basal dendrites. Arrows indicated typical FPs recorded at target deep layer 5. Bottom, current source density profiles corresponding to the laminar FPs evoked by stimulating at L5. By activating inputs targeting basal dendrites, the early, negative FP recorded in L5 was generated by direct inward current (that is, the sink, yellow/red). This feature was highly consistent among different animal subjects. (**b**) Top, FPs were recorded from rats undergoing 7 days of forelimb reaching task, followed by 3 days of rotarod running task. Bottom, potentiation of stimulation evoked-FPs slope when activating basal dendritic inputs in L5. Representative traces of the FPs on days 1 and 7 (pre- and post-training) are shown. All bars represent the mean±s.e.m (five rats). (**c**) Learning-associated shortening in delay of first reach attempt in sham-operated (black, five rats) and 6-OHDA lesioned animals (blue, five rats). Sham group: mean±s.d. of delay on day 1: 4.416±0.783 s, day 3: 3.362±0.588 s, *P*=0.038; day 7: 1.118±0.216 s, *P*=0.006, all compared to day 1, one-way repeated measures ANOVA, 5 rats; Lesioned group: mean±s.d. of delay on day 1: 3.663±0.676 s, day 4: 2.631±0.849 s, *P*=0.065; day 5: 2.376±0.778 s, *P*=0.048; day 7: 1.587±0.463 s, *P*=0.019, all compared to day 1, one-way repeated measures ANOVA, 5 rats. (**d**) Comparison of motor skill performance between sham-operated (black, five rats) and 6-OHDA lesioned (blue, five rats) animals. Data are represented as mean±s.e.m. In contrast to the sham group, the first reach success rate achieved after each day's training by the lesioned animals was not well maintained in the next day. (**e**) Comparing to sham-operated group (black, five rats), with local dopamine depletion restricted to M1 (blue, five rats), learning-induced potentiation of FPs could not be maintained. Data are represented as mean±s.e.m.

We speculated that the mechanism by which the motor representation is altered in M1 involves the induction and maintenance of experience dependent synaptic plasticity, as hinted by previous studies documenting highly dynamic spine remodelling processes in M1 during motor learning^29^,^30^. However, it is unknown whether these remodelling processes were associated with changes in the strengths of synaptic connections. We therefore examined the properties of motor training-induced synaptic plasticity occurring at the output layer neurons, by tracking the changes of FPs evoked and recorded at L5b (Fig. 7a, upper panel). We applied one-dimensional current source density (CSD) analysis to confirm the location of the current source underlying the FPs recorded (Fig. 7a, bottom panel). Locations of current sinks revealed that stimulation at L5b activated synaptic inputs mainly confined to the same layer, which is consistent with synaptic transmission occurring at the basal dendrites of L5b neurons.

Forelimb reaching motor training (days 1–7) was associated with a rapid and long-lasting potentiation of the evoked FPs in the first few days (Fig. 7b). The ceiling of this physiologically induced LTP was reached typically after day 4. Thus, the profile highly paralleled that of the behavioural learning curve of increasing first success rates (cf. Fig. 1f). When the animal switched to the rotarod running task from days 8 to 10, the evoked FPs were further potentiated (Fig. 7b, mean±s.e.m of potentiation on day 7: 125.8±1.3%, day 8: 128.2±1.7%, *P=*0.013; day 9: 129.9±1.5%, *P=*0.004; day 10: 130.3±1.0%, *P=*0.003, paired *t*-test, all compared to day 7, five rats). These results are consistent with the observation of an overall and persistent increase in basal dendritic spine density of L5 neurons with motor learning^51^ and strengthening of horizontal connections among them.

To explore potential relationship between single-unit activity and evoked FP, we performed spike sorting for recordings obtained from FP experiments (Supplementary Fig. 10a). As the FP experiments were carried out in animals with only linear electrodes implanted (Fig. 7a, top panel), we therefore only had a limited number of neurons available for this analysis (a total of 3 type 1 neurons and 11 type 2 neurons from 5 rats with sufficiently stable recordings as measured by high-quality spike sorting, highly preserved spike waveform and ISIH). Interestingly, for type 1 neurons, we identified a phase locking relationship whereas type 1 neurons exhibited a peak in peristimulus time histogram, and this phenomenon was apparent already in day 1 and persisted throughout 7 days of training (Supplementary Fig. 10b, upper panels). In contrast, type 2 neurons initially did not have a peak in their PSTH, but after training, they also exhibited locking of firing to FP (Supplementary Fig. 10b, lower panels). These findings suggest that there may be selective strengthening of recurrent connections among type 2 neurons.

### Critical role of dopamine in M1 for motor memory formation

Dopamine is known to be critical in synaptic plasticity^33^ and recent studies imply that the mesocortical dopaminergic innervation to M1 is essential for the acquisition of motor skills^34^,^35^. Thus, to further probe the mechanism of motor memory formation, we investigated the effect of dopamine denervation in M1 on both motor learning-induced LTP and changes in motor representation in our animals. Local dopamine depletion was achieved by 6-hydroxydopamine (6-OHDA) injection into M1 while noradrenergic terminals were spared by desipramine co-injection. The level and specificity of dopamine terminals depletion was verified immunohistochemically (Supplementary Fig. 11a–d).

Under local dopamine depletion, there was still learning-associated shortening of delay in first attempt during the 7 days of training. However, in contrast to sham-operated animals, for which the delay of first reach success trials was significantly shortened from day 3 onwards (Fig. 7c), significant shortening of response delay of lesioned animals was only observed from day 5 onwards (Fig. 7c), suggesting a degradation in learning performance after local cortical DA depletion. More strikingly, the first reach success rate achieved after training in a single day was not well maintained overnight, and led to repeated re-learning of the task in the next day (Fig. 7d). As such, the eventual first reach success rate after 7 days of training was substantially lower than that achieved by the sham control (mean±s.d. of first success rate in day 7 session 6: sham: 47.85±4.54%, five rats; lesioned: 38.3±6.48%, *P*=0.028, one-way ANOVA, five rats, Fig. 7d). Restoration of dopaminergic tone by administration of levodopa partially prevented degraded motor learning (Supplementary Fig. 11e, mean±s.d. of first success rate on day 7: 6-OHDA+vehicle: 34.8±4.2%, *n*=3 rats, 6-OHDA+L-DOPA: 41.9±2.9%, *n*=3 rats, *P*=0.035; sham: 48.1±2.2%, *n*=4 rats, *P*=0.021; all compared to 6-OHDA+vehicle group, Kruskal–Wallis H test), while M1 6-OHDA injection after completion of 7-day motor training did not impair further motor performance (Supplementary Fig. 11f), indicating that 6-OHDA injection did not exert its effects via impairing motor control *per se*. In parallel to these findings, we found that under dopamine depletion, the profile of motor training-induced LTP was severely disrupted. Despite that the training cohort still led to the potentiation of evoked FPs every day, significant depotentiation occurred overnight, resulting in the repeated cycles of potentiation/de-potentiation throughout the whole training period (Fig. 7e). These observations demonstrate an essential role of dopamine in consolidating newly potentiated synapses in L5b PNs.

**Figure 8:**
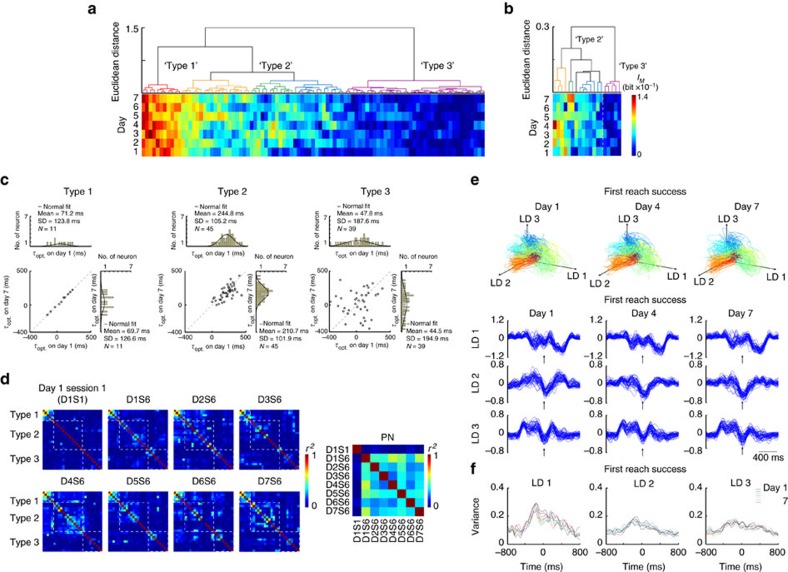
Neural dynamics of L5b PNs after dopamine depletion. (**a**) and (**b**) 95 PNs (**a**) and 19 INs (**b**) in L5b recorded from four rats with local dopamine depletion in L5 of M1 forelimb territory, were classified into subgroups by hierarchical clustering of single neuron *I*_*M*_ during motor learning, following the same method as shown in Fig. 3c. (**c**) Summary of training-dependent changes of the optimal time lag of *I*_*M*_ (*τ*_opt._) in three types of L5b PNs after dopamine depletion. Statistical quantification indicated that throughout 7 days' training, there was less consistent change and only a slight reduction of averaged *τ*_opt._ in type 2 PNs (type 1: *P*=0.681, *n*=11; type 2: *P*=0.042, *n*=45; type 3: *P*=0.852, *n*=39; paired *t*-test, 4 rats) compared with intact animals. (**d**) The pairwise cross-correlation matrix of 27 L5b PNs recorded from the same example rat shows that there was no emergence of consistent functional clusters after 7 days' motor training (cf. Fig. 5a). The averaged correlation values from the PNs of four lesioned rats from day 1 session 1(D1S1) to day 7 session 6 (D7S6) are shown on the right. (**e**) Top, single-trial neural trajectories of randomly selected first reach success trials (randomly selected 50 trials per day) performed by dopamine-depleted rat (27 L5b PNs recorded from a representative animal). The flow of time series is colour gradient coded, from blue (start) to red (end). Bottom, the same neural trajectories shown in three individual latent dimensional space (LD1-3). Arrow indicates ‘orient' position. Compared with intact animals, reproducible neuronal trajectories did not emerge during training, even in first reach success attempts (cf. Fig. 6b). (**f**) Analysis of the variance of the neural trajectories in LD1-3 after dopamine depletion (95 L5b PNs from four rats). Statistical quantification confirmed the lack of training-dependent reduction in variance even in first reach success trials at day 7 (cf. Fig. 6c).

If dopamine-mediated consolidation of synaptic plasticity is critical to motor memory formation, one would expect that the emergence of the task-related activities at the single neuron level as well as population level would be affected under dopamine depletion. Indeed, in these animals, while hierarchical clustering of the L5b PNs and INs based on *I*_*M*_ still succeeded in classifying sub-groups of neuronal clusters (95 PNs and 19 INs, Fig. 8a–c, four rats), the emergence of neurons with increase in *I*_*M*_ and shortening in *τ*_opt._, classified as type 2 neurons, was much less distinct (Fig. 8c, middle panel, mean±s.d. of *τ*_opt._=−23.4±74.2 ms, corresponding to an average of 11.4% reduction in 45/95 PNs). Analysis of pairwise correlation structure pattern revealed that while type 1 neurons maintained their structure of correlated activity throughout the training, the remaining neurons failed to form stable functional clusters (Fig. 8d). Furthermore, reproducible population dynamics failed to emerge under dopamine depletion, even during first reach success trials (Fig. 8e), as variance of neuronal trajectories remained unchanged throughout training (Fig. 8f and Supplementary Movie 3).

## Discussion

The M1 comprises neuronal populations distributed in different cortical layers. It was only until recent years that the neuronal layer-specific effects of learning, which are critical for understanding the neuronal circuitry underlying motor learning of M1, has started to be uncovered. However, due to its depth and therefore inaccessibility to optical functional imaging methods, the role of the output layer in M1 in the process of motor learning remains elusive. In this study, by making chronic extracellular recordings capturing the firing activities of L5b neurons over the entire training period, we have uncovered that these neurons could represent an adaptive, and also the ultimate, cortical sub-network critical for the generation of faster, more precise and consistent movements characteristics of learned skills.

Recording high-quality and reliable signals from large numbers of neurons is a prerequisite for investigating the temporal dynamics of neuronal populations. We compared two electrode designs, 16-channel microwire array and 4 tetrodes by tracking the signal reliability of recorded neural population over time. The stringent statistical quantifications demonstrated the viable chronic recording of both designs. We opted to employ single-channel recording, which allowed probing more samples of neurons simultaneously. This strategy was also suggested in other studies, which demonstrated that microwire array recording in region of moderate cell density, like the cortex and thalamus, maintained high neuronal yield from weeks to months^52^,^53^,^54^. It further justified the use of single-channel mode as an efficient way for chronic neuronal ensemble recordings^55^,^56^.

Over days of training, a substantial proportion of L5b neurons progressively changed from being non-informative about forelimb velocity and trajectory to possessing similar mutual information about motor behavioural outputs as neurons that exhibited clear movement encoding firing at the beginning of training. The decreased variance of these neurons during motor task execution with motor training also suggest that they become engaged in circuitry encoding the forelimb reaching task only after learning. This increase in proportion of L5b neurons, which are more informative of forelimb movement parameters, is similar to what has been shown recently for M1 L5a neurons^25^. Importantly, we have also identified the fine temporal scale of the refinement of motor coding, demonstrating a progressive shortening of the temporal lag on an order of tens of milliseconds with respect to when the instantaneous firing rate of single neuron is most informative of instantaneously forelimb velocity, and that this effect observed was not due to a shortening or reduced variance of motor task execution time with training. Therefore, apart from becoming more movement-encoding, task-recruited L5b neurons also become more time-locked to motor execution with a shorter time lag. These changes were accompanied by an increase in the collective predictive power of motor output by recruited L5b neurons, as forelimb movement velocity and trajectory could be decoded from the population activity of these neurons almost as accurately as from neurons that exhibited clear movement correlated firing early on in training, signifying their improved encoding of motor movement. Interestingly, we found that SVR decoding model trained on task-recruited neuronal population firing-motor data for a given day predicts previous day data with even higher accuracy than same day data. This may be due to increased tuning of task-recruited neurons, causing the optimized model on later day to be more informative and possess better noise tolerance, thereby allowing better generalization of SVR decoding model. It is difficult to be certain the exact projection type of these task-recruited PNs, but they are more likely corticostriatal neurons, while those PNs that were highly movement-encoding right at the beginning and exhibited higher spontaneous firing rates may correspond to corticopeduncular neurons^49^.

Motor learning-induced changes were not only reflected in the reduced firing variance and increased information content of single neurons during motor task execution, but also in the joint neuronal firing statistics, as a task-specific, stable correlation structure emerged and persisted among recruited L5b neurons during training. This observation shares similarity with M1 tongue area neurons, where neurons of similar response types became more correlated during training of a lick/no-lick task^22^. It is also consistent with the possibility that motor learning has led to the strengthening of task-relevant common inputs or horizontal connections among these L5b neurons. Overall, the neuronal population activity became less variable, as reflected in the more reproducible embedded three-dimensional neural activity trajectory during execution of forelimb reaching, especially during trials with successful reaching and grasping of food pellet on the first attempt, an effect that can be largely attributed to reduced firing variance of task-recruited neurons. Our data therefore suggest that characteristics of motor learning induced changes previously documented in both L2/3 and L5a of M1 (refs 22, 24, 25), the main input layers providing local excitatory drive^18^,^19^,^20^,^21^, are conveyed to, and therefore drive the reorganization of neural representations in the output layer L5b. Compared to L5b neurons that are highly movement encoding right at the beginning of motor training, task-recruited L5b neurons may represent those receiving more drive from L2/3 and L5a neurons whose firing have previously been shown to become more reproducible and exhibit increased motor outcome predictability^19^,^25^.

Altogether, the emergence of a larger pool of task specific, movement encoding L5b neurons that exhibit more correlated, less variable firing, with shorter and less variable time lead preceding motor execution, as well as more reproducible population activity, may be the neural substrate underlying enhanced precision of forelimb movement, as they may help to generate more temporally synchronous or amplified output signal from M1 to downstream circuitry for more precise motor control and execution.

What then underlies the motor representation plasticity observed in L5b? We attempted to provide direct evidence for the involvement of synaptic reorganization in L5b neurons. We revealed that evoked FPs in L5b were potentiated *in vivo* during the process of motor training. Although one cannot be certain of the exact pathways excited that resulted in the observed FPs, CSD analyses revealed the source of synaptic inputs being highly localized at L5b. Therefore, the most plausible explanation is that the current sinks correspond to trans-membrane synaptic currents in the basal dendrites of these neurons. The similarity between training-induced LTP and the performance of the animal in the motor task strongly implicates that the potentiation of synaptic inputs to L5b neurons contribute to the emergence of the motor memory, possibly among task-recruited neurons whose firing became phase-locked to evoked FP. Although spine remodelling at the basal dendrites of L5 neurons during motor learning is still uninvestigated, a recent Golgi staining study suggests that spine density on the basal dendrites of L2/3 neurons is increased during motor learning and maintained^51^, similar to the profile of training-induced LTP in the deep layer that we found. Our findings are also in line with observation that dopamine depletion in M1 impairs learning-induced spine formation and elimination^57^. However, our data do not exclude the possibility that PNs in other layers, for example, L2/3 and L6, contributed to the FPs recorded and the potentiation observed. On the other hand, our observation is at variance with imaging studies that demonstrated clustered spine formation on the apical dendrites of L5 neurons within the first few hours and days of motor skill training^28^,^29^, which is followed by elimination of old spines thus maintaining the overall spine density^28^,^30^. Thus, an important question remains to be determined is how changes in different functional inputs, as a result of spine remodelling, ultimately shapes the firing characteristics of L5b neurons.

Involvement of synaptic plasticity is further accentuated by the fact that in the absence of dopamine, LTP of FPs could not be maintained across days, suggesting resolving of newly potentiated synapses and therefore necessitating LTP to be established again in subsequent training sessions. Behaviourally, this manifests as repeated de-learning and re-learning of the motor task across days. An intact dopamine tone, however, does not appear to be necessary for the motor execution. Dopaminergic innervation from the midbrain ventral tegmental area^58^,^59^ to M1 may therefore be essential in consolidating potentiated synapses in the output layer of M1 and thereby motor memory.

## Methods

### Animals

Adult male Sprague Dawley rats weighing 280–300 g were housed under standard laboratory conditions (12-h light/dark cycle, and lights on at 7: 00) with water provided *ad libitum* and food restricted throughout the whole experiment period (at 85% of their *ad libitum* body weight). Experiments were performed with strict compliance to the university guidelines, with approval by the Animal Experimentation Ethics Committee of the Chinese University of Hong Kong.

### Functional mapping of the forelimb territory in M1

Intracortical microstimulation was applied to determine the functional map of the motor cortex contralateral to the trained forelimb. Rats were anaesthetized with chloral hydrate (400 mg kg^−1^, i.p.) and secured on stereotaxic apparatus (Narashige, Tokyo, Japan). After a linear incision in the scalp, the cerebral cortex was exposed by a unilateral craniotomy (8 mm anterior-posterior, and 0–5 mm lateral from the midline), keeping the dura intact. The intracortical microstimulation pattern was adapted from previous reports^60^. A low-impedance stimulating electrode penetrating the dura was positioned serially at 200 μm intervals, covering the entire exposed cerebral cortex. At each penetration site, the electrode was lowered deep into the M1 (1.5 mm dorsal-ventral). Electrical stimulation consisted of a train of ten 200 μs cathodal pulses delivered at 200 Hz from a constant current isolated stimulator (Model DS3, Digitimer Ltd, UK). At each site, pulse trains were delivered 2 s apart and the stimulating current was gradually increased (<100 μA) until evoked muscle contractions were observed and in a consistent manner while the animal was kept in the prone position. Sites with no muscle contraction evoked by the protocol were defined as nonresponsive. Areas with either distal (wrist/digit) or proximal (elbow) motor representations were regarded as the forelimb territory.

### Single-pellet forelimb reaching task

The rats performed single-pellet forelimb reaching which was adapted from Whishaw and others^61^. We designed an operant reaching chamber (35 × 30 × 25 cm), with a 1 cm wide slot in the middle of the front wall. Rats were allowed to reach a platform (4 cm high) through the slot and retrieve food pellets placed 1.5 cm away from the slot in the contralateral indentation (two indentations were spaced with 1.5 cm).

The animals were food-restricted to 85% of free-feeding body weight levels, which was maintained for the whole training period. One day before surgery (1-h pre-training habituation), animals were permitted to use either forelimb to retrieve centrally placed pellet. Once the animal made eight out of ten reaches with the same forelimb, this limb was defined as the animal's preferred limb^62^,^63^. After recovery from surgery, animals received 7 consecutive days of training on the preferred forelimb. Single food pellets of uniform size and shape were placed in the indentation contralateral to the preferred forelimb and animals were permitted to make reaching and grasping attempts. Rats received six training sessions every day, each session lasted 10 min with 5-min resting intervals. The whole training period lasted for 7 days. We set one high-speed video camera (80 fps) mounted above the platform to track forepaw trajectories for each reaching attempt and another video camera with lower frame rate (30 fps) heading upwards from chamber undersurface to track the forepaw position during inter attempt intervals. For each reaching attempt, forepaw trajectories were tracked automatically using the CinePlex behavioural research system (Plexon Inc.), which performed contour detection, image segmentation of forepaw and calculated the center of gravity for the forepaw as the positional measure. For accurate tracking, an arena of interest over the video image was drawn to reduce false object detections and exclude reflections outside the arena. The forelimb contour was tracked by applying background subtraction to compute the colour difference of the object with background frame by frame (see Fig. 1b, blue shaded area). The tracking window was repositioned automatically and a history of forepaw movement was used to predict its next position. To ensure quality of centre of gravity tracking, we visually verified the accuracies of forepaw outline tracing for individual videos and frames. During inter-trial intervals, animals almost stayed static holding food pellet. We performed manual tracking of forepaw trajectory frame by frame, and performed cubic spline interpolation to preserve forepaw tracking frequency for the whole recording session at 80 Hz.

An adaptation of the Whishaw reaching movement rating scale^64^ was used to assess forelimb reaching behaviour. A complete reaching attempt in general can be decomposed into six movement components: (1) ‘orient': the forelimb is lifted from the floor, reaching the slit opening of the chamber; (2) ‘advance': the forelimb moves forward towards the pellet; (3) ‘extend': the forelimb extends further, and the forepaw pronates over the target with the digits opened; (4) ‘grasp': the forepaw reaches the pellet and the digits close to grasp; (5) ‘retract': the forelimb is withdrawn through the slot, holding the pellet if successful; (6) ‘complete': termination of one reach attempt and, if successful, the food pellet is released for consumption. After consuming each pellet, a new food pellet would be provided once the rat reset its stance. A ‘first reach success' trial was defined as trials during which the animal completed the reaching attempt and consumed the food pellet successfully on the first reaching attempt, and a ‘first reach failure' trial was defined as the trial in which animal advanced the forelimb through the slot but missed the pellet on the first attempt, failed to grasp it, knocked it away or dropped it upon retraction. Behaviour improvement was quantified by: (1) delay of first attempt, defined as the time between provision of food pellet and the moment the forelimb reached the slit opening of the chamber; (2) first reach success rate, defined as the percentage of first reach success trials out of total number of reaching attempts. The temporal variability of movement execution was evaluated by: duration of extension (time from orient to extension), grasp (time from extension to grasp) and retraction (time from retraction to complete).

To quantify temporal evolution of trajectory variation with training, we performed DTW analysis to find the optimal mapping for individual reach trajectory to the reference expert trajectory, computed from the average of movement trajectories of 50 random first reach success trials from day 7 session 6, and then quantified the cumulative Euclidean distance deviation of individual trajectories from reference expert trajectory. Given two time sequences, the DTW algorithm first calculated the Euclidean distance between each point of two vectors and then searched for an optimal warping path with the smallest sum of distances between two vectors. The result was in arbitrary units, with higher distance values indicated greater deviation from the reference trajectory.

### Rotarod running task

After 7 days of forelimb reaching task, four rats were trained with an additional motor learning task on a rotarod treadmill (Rotaod ENV-576, Med Associates Inc., St Albans, VT, USA). The rotarod was set to accelerate with rotation speed increasing from 4 to 40 revolutions per minute over 300 s. Each trial ended when the rat fell off the rotarod or when 300 s was reached. The animal was then remounted on the rotarod for the next trial. Training lasted for 3 days. Each animal received six training sessions every day, and each session lasted for 10 min with 5-min resting intervals. The maximum time that the rat stayed on the rotarod, termed ‘latency to fall' (up to 300 s), was evaluated for each trial.

### Stimulating and recording electrodes implantation

Rats were anesthetized with chloral hydrate (400 mg kg^−1^, i.p.) and secured on stereotaxic apparatus (Narashige, Tokyo, Japan). For animals undergoing extracellular recording, two different electrode designs were used: (1) 16 single-ended microwires spaced 250 μm apart (Teflon coated stainless steel wire, 30 μm in diameter, arranged in a 4 × 4 array, Plexon Inc, Dallas, TX, USA); (2) four tetrodes spaced 250 μm apart (Teflon coated nichrome wire, 25 μm in diameter, California Fine Wire). Four wires were manually spun together in a braid and then insulated further with a polymide guide tube (80 μm inner diameter). Electrodes were implanted into L5b of the M1 forelimb territory (anterior-posterior (AP): 1.5 mm, medial-lateral (ML):±3.0 mm, dorsal-ventral (DV): 1.5 mm, Paxinos and Watson, 1986). To evoke FPs, a stimulating electrode targeting the same layer was implanted, and located 500–800 μm from the recording electrodes. To perform multi-laminar FP recording, 20-channel linear microprobe with 100-μm inter-site spacing (MicroProbes, MD, USA) was inserted orthogonal to the surface of the M1 forelimb territory (DV: from 0.2 to 2.1 mm). Three stainless steel screws firmly attached to the skull were employed for electrode anchoring, and one additional reference electrode was soldered to a screw attached to the skull at the lambda. The whole assembly was secured with dental cement.

### Focal denervation of meso-cortical dopamine projection

Rats were anesthetized with chloral hydrate (400 mg kg^−1^, i.p.). Thirty minutes before surgery, rats received an i.p. injection of desipramine hydrochloride (25 mg kg^−1^; Sigma) in order to prevent noradrenergic terminals from taking up 6-OHDA^65^. To selectively deplete meso-cortical dopamine innervation to the M1, 8 μg of 6-OHDA (Sigma, St Louis, MO, USA) dissolved in 2 μl of sterile 0.9% saline and 0.02% ascorbic acid (or 2 μl of 0.9% saline for sham injection) was injected into bilateral M1 forelimb territory at a rate of 1 μl min^−1^ respectively. The micro-syringe for injection stayed in the brain for 15 min in order to prevent backflow of solution. For levodopa (L-DOPA)-treated group, 6-OHDA lesioned or sham-operated animals received intraperitoneal injection of L-DOPA (Sigma) 30 min before behaviour training. L-DOPA was given at a dose of 15 mg kg^−1^ dissolved in vehicle solution (saline with 0.1% ascorbic acid), combined with the peripheral decarboxylase inhibitor benserazide-HCl (Sigma) at a dose of 15 mg kg^−1^.

### *In vivo* electrophysiological data acquisition

Extracellular FPs and multi-unit activities in M1 were recorded simultaneously by the OmniPlex Neural Data Acquisition System (Plexon Inc., Dallas, TX, USA), which were synchronized with the CinePlex Behavioral Research System (Plexon Inc., Dallas, TX, USA) that captured the forelimb movement trajectory via a high-speed camera mounted above the front wall of the operant chamber. Electrical stimuli were delivered by a constant current isolated stimulator (Model DS3, Digitimer Ltd). FPs were amplified (× 4,000), band-pass filtered (0.5–200 Hz, 4-pole Bessel) and sampled at 1 kHz. Continuous spike signals were amplified (× 4,000), band-pass filtered (300 Hz to 5 kHz, 4-pole Bessel) and sampled at 40 kHz. Spiking sorting was performed off-line to obtain single-unit neuronal activities.

### Single-unit spike sorting

Spike sorting was conducted by using the OFSS V3 software (Plexon Inc., Dallas, TX, USA), and based on a combination of automatic and manual sorting techniques as previously described^55^,^56^,^66^,^67^,^68^. In brief, after spike detection with a threshold set at three standard deviations above baseline signal, the K-means clustering algorithm and the valley seeking method was applied to produce an initial separation of waveforms into individual clusters. Each cluster was then checked manually to ensure that the cluster boundaries were well separated and spike waveforms were repeatable. Spikes generated by the same neuron formed a discrete, isolated cluster in principal component (PC) space distinct from each other, and each discrete cluster was termed a ‘unit'. To objectively quantify the overall separation among multiple clusters in a given recording channel, four statistical parameters were calculated for clusters in the first two dimensions of PC space, which included the parametric F statistic of MANOVA (-logarithm), the J3 statistics (dividing by number of units), the Davis-Bouldin (-DB value) validity index and Dunn validity metric^55^. The null hypothesis in the MANOVA is that all clusters share the same underlying statistical distribution in PC space. A small *P* value indicates that each of the unit clusters has a statistically different location in PC space, and thus the clusters are statistically well separated. J3 is a measure of the ratio of between-cluster to within-cluster scatter; DB accesses the ratio of the sum of within-cluster scatter to between cluster separations; the Dunn index depicts the ratio of between-cluster distance to diameter of cluster. Low value of DB and high value of F, J3 and Dunn index would define well-separated clusters.

The signal-to noise ratio for a channel was calculated as the ratio of the single-unit waveform amplitude to the average noise amplitude. To quantify single-unit isolation quality, we employed multiple quantitative measures, including ID, L-ratio^39^,^40^, and refractory period reflected in ISI histograms. For units recorded by microwire array, energy, peak, valley, and the first three PCA coefficient were calculated for each unit spikes (six feature quantities); for units recorded by tetrode, energy and the first PCA coefficient were calculated for each unit spikes (eight feature quantities). The multi-feature quantities defined each spike as a point in a high dimensional space. Both ID and L-ratio employ the Mahalanobis distance for quantifying the location of spikes with respect to the centre of the corresponding cluster. ID was defined as the radius of the smallest ellipsoid from the cluster centre containing all the cluster spikes and an equal number of noise spikes. Thus ID estimated how distant the cluster spikes are from the other spikes recorded on the same electrode. L-ratio measured the amount of noise spikes observed in the vicinity of a given cluster; thus a low value indicates that the cluster was well separated from other spikes recorded on the same electrode. Units with signal-to noise ratio>4, ID≥15, L-ratio≤0.2 and 99.5% events with ISI>2 ms were considered putative single-units (cf. acceptable values of ID and L-ratio reported in previous studies^41^,^42^,^43^). No systematic differences in these measures existed between the three types of neurons reported in the study (mean±s.d. of averaged ID for type 1 neurons: 49.03±17.31, *n*=14, type 2 neurons: 47.85±17.68, type 3 neurons: 49.61±19.39, *P*=0.847, one-way ANOVA; L-ratio for type 1 neurons: 0.0307±0.0249, type 2 neurons: 0.0332±0.0261, type 3 neurons: 0.0351±0.0282, *P*=0.723, one-way ANOVA). Units recorded on different channels of microwire array were considered distinct neurons as the spacing between neighbouring channels was 250 μm. We further inspected unit pairs, aided by auto-correlogram and cross-correlogram as additional separation tools.

### Tracking of neurons over multiple days

The first two dimensions of each single-unit in PCs space obtained from recordings of each day were rendered as ellipsoids centred around cluster mean with three standard deviations around and stacked into cylinders with time as the third dimension. Straight cylinders indicated that single-unit isolation remained stable and there was no significant variation of clustering in PCs throughout recording sessions over the training period. We employed four measures to evaluate conservation of single-unit spike waveform and firing characteristics for classifying whether a given single-unit cluster correspond to stable tracking of the same neuron over the training period. The similarity of spike waveform shape was quantified as the maximum time-shifted linear correlation coefficient (*Max r*) between averaged spike waveform on a given day and day 1, and the resulting coefficient was Fisher-transformed to make it more normally distributed. The normalized peak-to-peak amplitude difference (Δ*P*_amp_) was computed as the ratio of spike amplitude change on a given day relative to day 1. To characterize single-unit firing property, we computed log-scaled ISIH from 0.5 to 10^5^ ms (in 100 bins) and log-scaled autocorrelogram (±100 ms in 100 bins), and then calculated the Kullback–Leibler (KL) divergence between pairs of normalized ISIHs or autocorrelograms which were log-transformed to approximate Gaussian distributions. Based on the dataset recorded within 30 min (two sequential 10-min recording session separated by a 5-min interval), we then calculated a set of synthetic ‘true positive' values from recordings that were presumably stably corresponding to the same neuron (microwire array: 247 units from five rats; tetrodes: 63 units from five rats), and a set of ‘true negative' values from recordings on different electrode channels on microwire array (as neighbouring electrodes on microwire array were separated by 250 μm and we assumed even adjacent electrodes would not record spikes from the same neurons), or distinct tetrodes (microwire array: 2,801 true-negative values from 247 units from distinct electrodes with neighbouring electrodes at 250 μm space apart, five rats; tetrode: 140 true-negatives values computed from 63 units from distinct tetrodes, five rats). Pairs of the four scores were combined and fitted with two-dimensional Gaussian mixture distribution and quadratic discriminant analysis was applied to determine the optimal quadratic decision boundary between true positive and true negative clusters. For each Gaussian mixture distribution, we employed criteria as described in a previous study^69^ and calibrated the decision boundary of quadratic classifier to produce a 5% error rate in the known ‘true negatives' cluster. Considering the ‘true-positives' dataset likely underestimate the natural variability of stable units over days, we controlled the maximum allowable false positive rate and set the conservative separation threshold as the mean plus 3s.d. of the true-negative score. Recordings of a single-unit were classified as stably corresponding to the same neuron only if the joint distribution of the criteria falls out of 3 s.d. from mean of distinct units and not classified as from distinct units by QDA throughout the 7 days. The *Max* *r*–ISIH dissimilarity score joint distribution as the optimal discrimination model because these were the two most informative features that gave the lowest BIC and AIC (microwire array, AIC: 4.84 × 10^−3^, BIC: 4.90 × 10^−3^; tetrode: AIC: 4.84 × 10^−3^, BIC: 5.13 × 10^−3^). For each day's data, we estimated the false positive rate as the percentage of single-unit recordings from distinct neurons deemed to be stable tracking of the same neuron, and false negative rate as the percentage of single-unit recordings from same neuron deemed to be distinct. As each electrode recorded from different units, we used the percentage of units from distinct electrodes that satisfy the stability criterion as an estimate of the chance of falsely positives. We assumed unit recorded in two continuously sessions (10 min each) were stable, and used the percentage of actual same unit that failed to satisfy the stability criterion as an estimate of the chance of falsely negatives. Criteria of sorting quality for all included single-units remained stable over the 7-day training period (Fig. 2f, mean±s.d. of ID on day 1: 48.68±18.28, day 7: 49.37±18.53, *P*=0.391; mean±s.d. of L-ratio on day 1: 0.033±0.027, day 7: 0.034±0.021, *P*=0.663, one-way repeated measures ANOVA, *n*=158; tetrode, Supplementary Fig. 2j, mean±s.d. of ID on day 1: 61.77±40.38, day 7: 60.14±40.31, *P*=0.315; mean±s.d. of L-ratio on day 1: 0.033±0.025, day 7: 0.032±0.026, *P*=0.346, one-way repeated measures ANOVA, *n*=41).

### Cell type identification

Based on the offline sorting results, putative pyramidal projection neurons (PNs) and INs were categorized based on three electrophysiological properties: spike valley-to-peak width (*t*, ms), valley-to-peak amplitude ratio (*v*/*p*) and mean firing rate^68^. Automatic clustering of these average waveforms from individual cells by using a k-means algorithm discriminated two groups of cells.

### Analysis of task correlates of single neuron activity

For individual unit, spike trains were discretized (bin size: 12.5 ms) and PETHs were calculated in relation to forelimb movement, from 6 s before forelimb ‘orient' position until 1 s after this event, or from 1 s before food provided until 6 s after this event. PSTHs were then smoothed with 5-point Gaussian filter, z-scored and plotted in each learning session (both first reach success and failure trials). The significant changes in firing rate were compared to pre-trial activities by bootstrapping. If the *P* value of firing rates falls below 0.01 for 5 or more successive time bins, the neuron was quantified as behaviour-correlated and the time point where the *P*-value first falls below 0.01 was termed time until divergence. The time interval between time until divergence and forelimb ‘orient' gives an estimate of the response times of single neurons.

### Estimation of time-lagged mutual information (*I*_*M*_)

To quantify the information of forelimb instantaneous velocity carried by single-unit activity, we estimated the time-lagged mutual information^70^ *I*_*X,Y*_ (*τ*) between single-unit spike train and forelimb movement instantaneous velocity. Formally, let *X* denote forelimb velocity and *Y* denote single-unit spike train recorded simultaneously, the mutual information of *X* and *Y* is defined as:


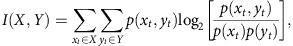


where *p*(*x*_*t*_, *y*_*t*_) is the joint probability distribution function of *X* and *Y*, *p*(*x*_*t*_)and *p*(*y*_*t*_) are the marginal probability distribution functions of *X* and *Y* respectively, and carries unit of bit with logarithm base 2 used. By introducing a time lag *τ* in either one of the variables, time-lagged mutual information can be given in a directional sense and computed as:


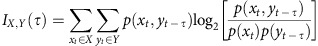


in which *I*_*X,Y*_ (*τ*) is quantified as a function of *τ*. Single-unit activity was discretized in time (bin size=12.5 ms) and shifted backward or forward bin by bin (*τ* ranged from −500 to 500 ms), and the optimal time lag *τ*_opt._ was defined as the value of *τ* where *I*_*X,Y*_ (*τ*) attains maximum (*I*_*M*_).

To test the null hypothesis that the observed pattern of *I*_*M*_ evolution with time arose by chance as a result of firing rate fluctuation independent of forelimb action, for each neuron we bootstrap resampled (10^7^ times) the firing rate data of 5 min recordings to obtain estimates of *I*_*M*_ distribution under the null hypothesis. Statistical significance was determined as *I*_*M*_ greater than 99% of *I*_*M*_ distribution under null hypothesis (corresponding to *P*<0.01).

To account for the across-trial variability of task execution time and behaviour kinematics causing possible apparent shortening of *τ*_opt._, we specifically extracted individual forelimb trajectory during periods of ±1 s when forepaw was within experimental arena and controlled for the variability by performing DTW to find the optimal mapping of individual trial trajectories to the reference expert trial, and applied the optimal trajectory warping path to time series of neural activity and the instantaneous velocity. The time-lagged mutual information was then re-calculated.

### Neural variability evaluated by mean-matched FF

We employed the mean-matched FF for quantification of single-unit firing variance as previously reported using the Variance Toolbox for MATLAB^48^. Spike counts were computed in a 50 ms sliding window in 10 ms steps. The firing rate was calculated as the spike count in each time bin divided by the length of the window. FFs for each time bin of single-unit activities covering ±1 s relative to the time of forelimb grasping were calculated. The statistical significance of FF was evaluated by comparing the overall FFs in three key epochs: pre-grasp (−1 to −0.5 s before grasp), during grasp (−0.25 to −0.25 s) and post-grasp (0.5 to 1 s).

### Hierarchical clustering analysis

Hierarchical clustering was performed using the MATLAB Statistics Toolbox function *clustergram* based on the Euclidean distance metric. For each neuron, the maxima of time-lagged mutual information *I*_*M*_ across 7 training days generated one 7-dimensional vector, and the Euclidean distance between pairs of neuron's *I*_*M*_ vector was calculated. According to the matrix of distance, an iterative agglomerative procedure, Ward's minimum variance method, was used to combine neuron into groups such that at each stage the total number of groups was reduced by merging groups whose combination gave the smallest possible increase in the within-group sum of squared deviation. The step was repeated until only one cluster remained. Putative PNs and INs were identified first and classification procedure was performed for PNs and INs separately. The optimal number of clusters based on *I*_*M*_ was evaluated by the MATLAB Statistics Toolbox function *evalclusters* based on the Calinski–Harabasz clustering criterion. Three main types of PNs (type 1, 2, 3) were determined by statistically estimating the training correlated changes of optimal time lag (*τ*_opt_. To explore for any signs of evolution of neural responses within 1 training day, we ordered three types of neurons in the same hierarchical sequence and recomputed *I*_*M*_ and *τ*_opt._ from sessions 1 to 6 within the first training day.

### Evaluation of neuronal activity-correlation pattern

To evaluate the joint neuronal firing statistics during motor learning, we calculated pairwise cross-correlation across all pairs of neurons' spike trains in a given time period. The time period was restricted to from ‘food provided' to ‘complete' state in single success trials. Spike trains were binned (12.5 ms) to get the instantaneous firing rates, which were then convolved with a Gaussian filter for smoothing. The squared cross-correlation coefficient (*r*^2^) between pairs of neuron's firing activity was calculated to generate the correlation matrix for each training session. Neurons were ordered following the ranks from hierarchical clustering of *I*_*M*_. The similarity of correlation matrices across different training days was assessed using the correlation coefficient of off-diagonal elements. To account for the possibility that the emerged correlation reflects merely changes in across-trial variability of forelimb reaching kinematics, we also computed correlations for type 1 and type 2 neurons using activities recorded when the animal was not executing the task.

### Decoding of forelimb movement information by SVR

SVR^71^ was employed to predict instantaneous forelimb velocity by neuronal activities, using the LIBSVM toolbox V1.04 in MATLAB^71^. To obtain the optimal preceding time window of each neuron's activity for decoding instantaneous velocity, single neuron SVR decoding was first performed. Neural activity was discretized in time bins of 12.5 ms corresponding to the frame duration of the video camera for behavioural recording. Given *N* neurons, let *x*_*i*_(*t*) denote the discretized firing rate time series of neuron *i* and let *y*(*t*) denote forelimb velocity. The optimal time lag for neuron *i* was found as the value of 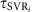 which allowed the best approximation of *y*(*t*) for all *t* by the SVR mapping 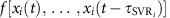, where 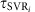 are multiples of discretization time bin (12.5 ms). While *τ*_opt._ represents the time difference that maximizes mutual information between instantaneous firing rate and forelimb velocity, for SVR prediction of forelimb velocity, firing before time window covered by *t*_0_ to *t*−*τ*_opt._ can also be informative about forelimb velocity. To maximize decoding/prediction accuracy, one would need to include all time points that jointly give the best information content. Consistent with this, 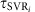 for individual neurons are invariably greater than or equal to *τ*_opt._ (Supplementary Fig. 6d).

After performing single neuron SVR decoding, we then performed SVR decoding based on neuronal population activity. For each neural population, the maximum of 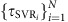, denoted 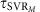, was chosen as the time lag for the population decoding of forelimb velocity, to cover a time window that includes all neural activity that maximize decoding performance for individual neurons. To perform neuronal population decoding of forelimb velocity, the goal of SVR algorithm was to find an optimal hyperplane 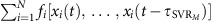 that best approximated *y*(*t*) for all *t*; hence the input vector had dimensionality of 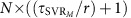, where *r* corresponds to the frame duration (12.5 ms). To obtain a good model, fivefold cross-validation was carried out and a set of parameters that minimized the mean squared error was selected. Specifically, SVR used a parameter *v*∈(0,1] to control the number of support vectors. Grid search function in Libsvm was used to find the optimal parameters cost *c* (−5≤log_2_*c*≤10)) and gamma (*g* −10≤log_2_ *g*≤5) for the radial basis function kernel. Prediction accuracy was quantified by squared correlation coefficient and mean squared deviation between predicted and actual behaviour output.

### Dimensionality reduction of population neural responses

GPFA^50^ was used for extraction and visualization of the neural population activity trajectory of individual trials. Briefly, this method worked by performing smoothing of spike trains and dimensionality reduction simultaneously within a common probabilistic framework. We performed the analysis by using GPFA MATLAB toolbox^50^. It assumed that the observed activity neuronal population was a linear function (plus noise) of a low-dimensional neural state, whose evolution in time was well described by a Gaussian process, and the probabilistic framework allowed good resolution of subtle neural dynamics. To minimize the potential contribution of reduced behavioural kinematics variance to neural variance changes with training, we selected first reach success trials with forelimb trajectories close to expert trajectory from each individual days of training (criteria: 30 randomly selected first reach success trials with trajectory deviation within mean±s.d. of cumulative Euclidean distance after DTW). Neural dynamics during these behavioural-variation consistent first reach trials, as well as first reach failure trials (30 randomly selected trials for each day) were visualized by performing GPFA on population neuronal activities (three types of neurons pooled together or separately), consisting of discretized spike counts (non-overlapping 10 ms bins, then square-rooted) from 800 ms before to 800 ms after the forelimb ‘orient' states. Three latent dimensions that resulted in good separation of the data points were shown in the figures.

### One-dimensional current source density analysis

CSD^72^, distribution *C* is a quantity that gives the location of current sources (cationic flux from the intracellular to the extracellular space) and sinks (cationic flow from the extracellular to intracellular space to achieve electroneutrality) that give rise to the stimulation evoked FPs. FPs (Φ) is related to *C* by


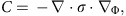


where ∇ denotes gradient operator and *σ* is the conductivity tensor. In a volume element, *C* is positive if outward currents dominate (source), and negative if inward currents dominate (sink).

In laminar cortical structures, which are isotropic and homogeneous, simultaneous excitation of a cortical lamina results in extracellular current that flows invariant in the direction perpendicular to the cortical plane. In this case the one-dimensional CSD analysis is applied and could be computed by


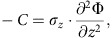


where *z* is the direction perpendicular to the cortical plane along which voltage gradient is sampled.

The MATLAB GUI toolbox CSDplotter-0.1.1 (ref. 73) was applied to produce continuous 1D-CSD based on the spline inverse CSD (iCSD) method, which assumed the current distribution to be continuously varying in depth (*z*) but homogeneous in the *x,y* dimensions and used standard electrostatic theory to establish a forward model matrix *F* between the measured FPs from *N* recording sites and CSD distribution. The inverse of the matrix *F*^−1^ was then used to estimate the CSD as 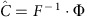. With *N* separate measurements of the FPs Φ, the smoothly varying CSD along *z* dimension was constructed by cubic spline interpolation.

### Evaluation of learning related synaptic plasticity *in vivo*

Ten days after surgery, synaptic inputs targeting basal and apical dendrites were assayed by stimulating the deep L5 and superficial L1 respectively. FPs evoked by monophasic electrical pulses (100 μs pulse width) at increasing intensities (40, 60, 80, 100, 200, 400, 600, 800, 1,000 μA) were recorded, with an inter-pulse interval at 30 s. Twenty continuous sweeps of FPs amplitude measurements were averaged to obtain the input–output curve. We obtained linear regression of the input–output curve and chose the test stimulation intensity for evaluation at between 25 and 75% (usually at 50%) of the intensity that evoked the maximal FPs amplitude. To monitor the stability of the baseline of evoked FPs, the amplitude of the FPs at the test stimulation intensity was evaluated twice per day over 5 days.

To investigate motor learning-related synaptic plasticity occurring at L5b in behaving rats, stimulations at the test stimulation intensity were delivered every 20 s in turn during the 5 min inter-session resting interval. Fifteen continuous sweeps of FPs amplitude were averaged, and the level of learning-related synaptic potentiation was calculated as the increment of the evoked FPs initial slope and amplitude relative to the baseline level^74^.

### Histological analysis

Rats were deeply anesthetized with chloral hydrate (400 mg kg^−1^, i.p.) and perfused transcardially with 300 ml ice-cold PBS, followed by 200 ml 4% paraformaldehyde. The brain was then quickly removed from the skull and post-fixed in the same fixative for 24 h. The fixed brain was transferred to 30% sucrose solution in PBS with pH 7.4 until it sank, then embedded in a tissue-freezing medium (OCT) and stored at −30 °C. Coronal sections (20 μm) of the M1 were cut by a freezing microtome (CS1031L9705, Shandon, UK).

For immunohistochemistry, the primary antibodies used included anti-Cux1 (CDP, rabbit, 1:100, Santa Cruz Biotechnology, catalogue No: M-222), anti-VGlut2 (Guinea Pig, 1:5,000, Millipore, catalogue No: AB2251), anti-TH (rabbit, 1:500, Millipore, catalogue No: AB152), and anti-NeuN (mouse, 1:500, Millipore, catalogue No: MAB377). Sections were incubated in primary antibodies at 4 °C overnight, and stained with the secondary antibodies. To identify the laminar structure of the M1, the layer specific marker Cux1 (labels L2/3) (ref. 75) and VGlut2 (labels L1, L2/3b, L5b) were doubly stained using Alexa Fluor 488 and 546 conjugated secondary antibodies (Invitrogen Corporation). The exact stimulating and recording site were confirmed by verifying the depth of electrode track with reference to the thickness of each cortical layer.

### Statistics

No randomization method was used and data were not analysed blindly. Data are presented as mean±s.d./s.e.m., unless otherwise specified. Paired *t*-test, one-way repeated measures ANOVA, least squares regression, Pearson's correlation test was used for statistical evaluation. Methods used for each analysis are mentioned in the main text and corresponding figure legends. No statistical methods were used to pre-determine sample sizes. The sample sizes in this study are in general similar to those employed in the field. Unless specified otherwise, the variance was similar between groups that were statistically compared.

### Data availability

The data that support the findings of this study are available from the corresponding authors on request.

## Additional information

**How to cite this article:** Li, Q. *et al*. Refinement of learned skilled movement representation in motor cortex deep output layer. *Nat. Commun.* **8**, 15834 doi: 10.1038/ncomms15834 (2017).

**Publisher's note:** Springer Nature remains neutral with regard to jurisdictional claims in published maps and institutional affiliations.

## Supplementary Material

Supplementary Information Supplementary Figures

Supplementary Movie 1 Kinematics of first reach success attempt and population neuronal trajectories. Left-top, an example of first reach success attempt captured by high-speed cameras and tracking of the center of gravity of forepaw (red dot). The movie was captured during the last session of training on day 7. Left-bottom, plots of corresponding two-dimensional (X-Y) forelimb displacement and instantaneous velocity. Time '0' was set at the forelimb 'orient' status. Right, single-trial neural trajectories in the first three orthonormalized latent dimensional space, extracted from population neural activities during 50 repeated first reach success trials by applying GPFA. The movie is slowed to approximately one-tenth of the real speed.

Supplementary Movie 2 Kinematics of first reach failure attempt and population neuronal trajectories. Left-top, an example of first reach failure attempt by the same rat shown in Supplementary Movie 1. The movie was captured during the last session of training on day 7. Left-bottom, plots of corresponding two-dimensional (X-Y) forelimb displacement and instantaneous velocity. Time '0' was set at the forelimb 'orient' status. Right, single-trial neural trajectories during 50 repeated first reach failure trials by applying GPFA. The movie is slowed to approximately one-tenth of real speed.

Supplementary Movie 3 Kinematics of first reach success attempt and population neuronal trajectories after focal dopamine depletion in M1. Left-top, an example of first reach success attempt after focal dopamine depletion in M1. The movie was captured during the last session of training on day 7. Left-bottom, plots of corresponding two-dimensional (X-Y) forelimb displacement and instantaneous velocity. Time '0' was set at the forelimb 'orient' status. Right, single-trial neural trajectories extracted from population neural activities of a 6-OHDA lesioned rat during 50 repeated first reach success trials. The movie is slowed to approximately one-tenth of real speed.
