# Supplementary material for: Refinement of learned skilled movement representation in motor cortex deep output layer
Source: Nat Commun. 2017 Jun 9;8:15834. doi: 10.1038/ncomms15834 (PMC5472789; doi:10.1038/ncomms15834)
Supplement: Supplementary Information — Supplementary Figures [file ncomms15834-s1.pdf]

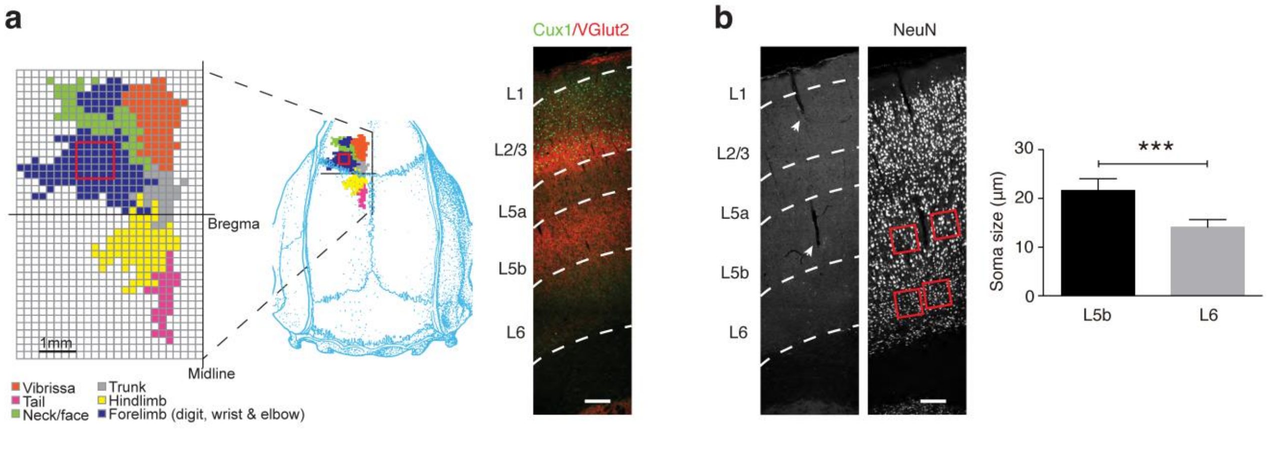

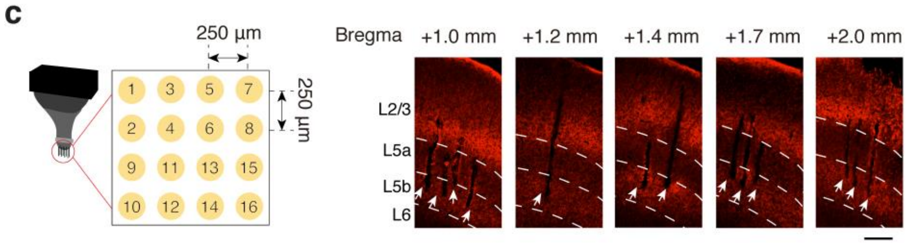

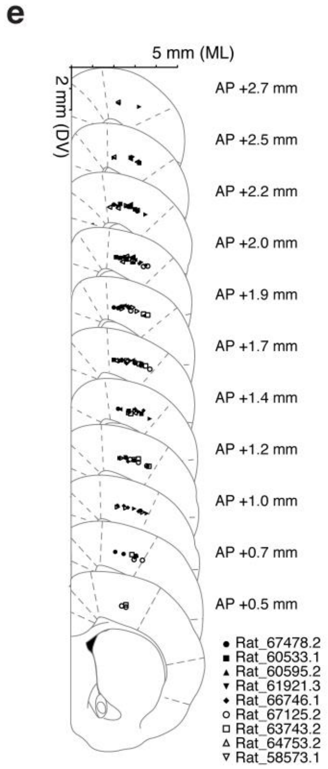

d

Estimated electrode positions targeting M1 L5b (AP from begma in mm)

Channel	Rat_67478.2	Rat_60533.1	Rat_60595.2	Rat_61921.3	Rat_66746.1	Rat_67125.2	Rat_63743.2	Rat_64753.2	Rat_58573.1
Ch 1	2.2	2.5	2.7	Out of range	2.0	Out of range	1.9	Out of range	2.5
Ch 3	2.0	2.5	2.5	Out of range	2.0	2.0	1.9	Out of range	Out of range
Ch 5	2.0	Out of range	Out of range	2.5	2.0	2.0	2.0	Out of range	2.7
Ch 7	2.2	Out of range	2.5	2.7	Out of range	2.0	Out of range	2.2	2.5
Ch 2	Out of range	2.2	2.2	Out of range	Out of range	1.7	1.7	Out of range	2.2
Ch 4	1.9	2.2	2.0	2.0	1.7	1.7	1.7	1.9	2.2
Ch 6	1.9	2.2	2.0	2.2	1.7	1.7	1.4	1.9	2.2
Ch 8	1.9	Out of range	2.2	2.2	1.7	1.9	1.7	1.9	2.2
Ch 9	Out of range	Out of range	1.4	1.9	1.4	1.2	1.2	Out of range	2.0
Ch 11	1.2	2.0	1.4	1.7	1.4	1.2	1.2	1.4	2.0
Ch 13	1.2	2.0	1.7	1.9	1.2	1.2	1.2	1.4	2.0
Ch 15	1.4	2.0	1.7	1.9	Out of range	Out of range	Out of range	1.2	1.9
Ch 10	1.0	1.4	1.0	1.2	1.0	0.7	Out of range	1.0	1.4
Ch 12	0.7	1.4	1.0	Out of range	1.0	0.7	Out of range	0.7	1.7
Ch 14	0.7	1.7	1.2	1.2	1.0	0.5	0.7	1.0	1.7
Ch 16	0.7	1.7	1.2	1.4	1.0	0.5	0.5	1.0	1.7

Supplementary Figure 1 Functional map of the M1 and histological verification and reconstruction of electrode placement in L5b of M1. (**a**) Left, cumulative distribution of functional map of motor cortex contralateral to the trained forelimb in 6 naive animals using standard intracortical microstimulation technique. Each stimulation site was approximately 200 μm apart (grid size). The caudal and rostral part of forelimb territory (purple) is separated by a band of neck/head representations (green). The red square indicates the position of electrode implantation, targeting caudal forelimb area (anterior-posterior (AP): +1.5 mm, medial-lateral (ML): ± 3.0 mm, dorsal-ventral (DV): 1.5 mm). Right, schematic of cortical layers depicting the laminar specific expression of biomarkers in cortex. Cux1-immunopositive neurons (green) are located in L2/3. L1, L2/3b, and L5b are more immunopositive for VGlut2 (red). Scale bar = 200 μm. (**b**) Left, bright field image of coronal M1 section with electrode track. Two white arrows mark electrode tracks targeting superficial cortical layer and deep L5b respectively. Middle, the same slice shown on the left, stained with neuronal biomarker NeuN. Lamination appeared intact. Right, soma size evaluation based on NeuN positive neurons in L5b and L6. (L5b: 21.6 ± 2.4 μm (mean ± s.d.), measured from 561 cells from around each tip (two red squared zones in L5b); L6: 14.1 ± 1.5 μm (mean ± s.d.), measured from 525 cells from > 200 μm from deepest point of each electrode tip (two red squared zones in L6). Scale bar = 200 μm. (**c**) Left, electrode configuration. 16 channels in 4 × 4 array, consisting of 35

μm electrodes spaced at 250 μm intervals. Right, coronal view of VGlut2-stained sections with electrode tracks (arrows) targeting L5b of M1 (Rat_66746.1). The brain tissue in the vicinity of the electrode track appeared largely intact. Scale bar = 300 μm. (**d**) Positions of all recording channels from 9 rats used for extracellular recording experiment. More than 20 single units from M1 L5 were isolated from rats highlighted in red and data from these animals were included for neural population analysis. Green colored boxes indicate channels with high signal-to-noise ratio spikes included for further analysis. Yellow color marks channels from which spike recording failed. (**e**) Reconstruction of all electrode placements in L5b of M1 from 9 rats shown in **(d)**. Recording depths were determined from the deepest end of electrode track. Different symbols are assigned to individual rat.

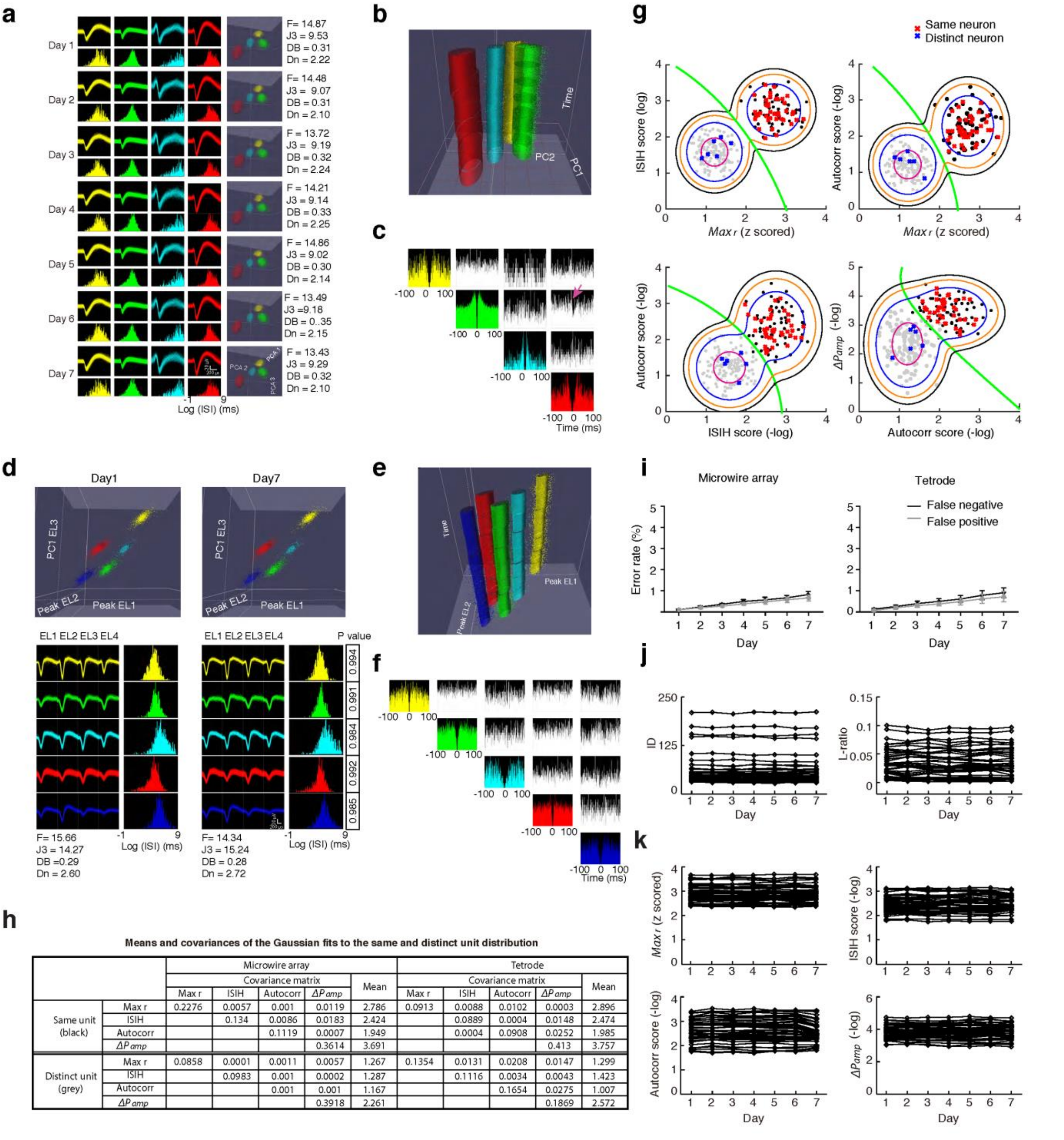

Means and covariances of the Gaussian fits to the same and distinct unit distribution

		Microwire array					Tetrode				
		Covariance matrix				Mean	Covariance matrix				Mean
		Max r	ISIH	Autocorr	ΔP_{amp}		Max r	ISIH	Autocorr	ΔP_{amp}	
Same unit (black)	Max r	0.2276	0.0057	0.001	0.0119	2.786	0.0913	0.0088	0.0102	0.0003	2.896
	ISIH		0.134	0.0086	0.0183	2.424		0.0889	0.0004	0.0148	2.474
	Autocorr			0.1119	0.0007	1.949		0.0004	0.0908	0.0252	1.985
	ΔP_{amp}				0.3614	3.691				0.413	3.757
Distinct unit (grey)	Max r	0.0858	0.0001	0.0011	0.0057	1.267	0.1354	0.0131	0.0208	0.0147	1.299
	ISIH		0.0983	0.001	0.0002	1.287		0.1116	0.0034	0.0043	1.423
	Autocorr			0.001	0.001	1.167			0.1654	0.0275	1.007
	ΔP_{amp}				0.3918	2.261				0.1869	2.572

Supplementary Figure 2 Comparison of single microwire array and tetrode regarding long-term stability of isolated single units recorded over 7 days. (a)-(c) Identical arrangement as in **Fig. 2a-c** for another single microwire array channel, with three putative pyramidal neurons (yellow, blue, red) and one interneuron (green). Short latency suppression in the cross-correlogram (arrow) indicated a presumably monosynaptic, inhibitory interaction. **(d)-(h)** Long-term stability of multiple single units recorded by tetrode. **(d)** Top, 5 color-coded single units recorded from a single tetrode remained stable from day 1 to day 7, revealed by measuring the spike peak recorded from three tetrode channels (EL1-3, 2 minutes recording before training). Bottom, for each unit the superimposed spike waveforms are shown on the left panel and the inter-spike-interval (ISI) histogram shown in the right panel. **(e)** Long term stability of identified single-unit clusters in principal components space shown in **(d)**. **(f)** Auto-correlograms of the 5 well isolated units and their cross-correlogram (white). **(g)** Combining multiple similarity scores with quadratic classifier. 4 similarity scores included: spike similarity ($Max\,r$), ISIH similarity score,

autocorrelation histogram similarity score, and spike peak-to-peak difference (ΔP_{amp}), between spikes recorded from same neuron (black dot, acquired in difference sessions on the same day) or distinct ones (grey dot, spikes acquired by difference channels simultaneously) on the same day. Contours of the Gaussian mixture distributions fitted for are shown (50%, red; 95%, blue; 99.9%, orange; 99.97%, black). **(h)** Mean and covariance matrices for the fitted Gaussian mixture distributions for both same and distinct units from microwire array and tetrodes recordings. (**i**) Cumulative false negative and false positive rates over recording periods. **(j)** Cross-day stability of single unit isolation quality assessed by ID and L-ratio (n = 41) for included units shown in red in (**g**). **(k)** Cross-day stability of four cluster similarity scores.

a

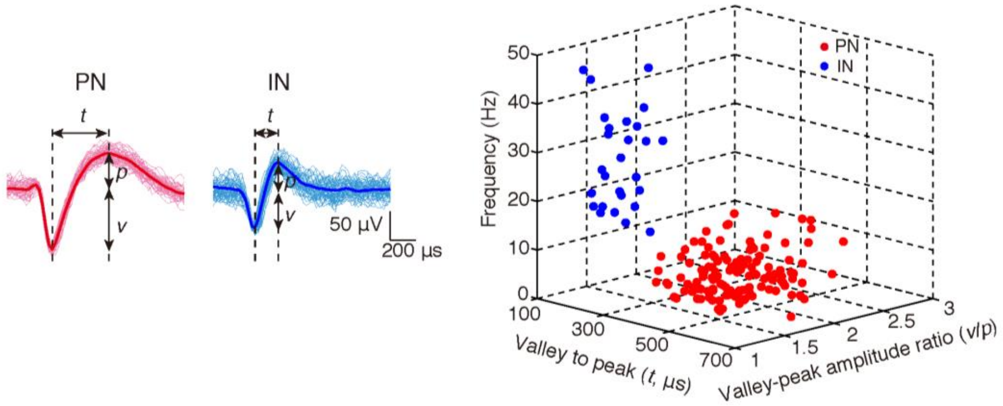

b

	Pyramidal neuron (PN)	Interneuron (IN)
Intact (5 rats)	131 (82.9%)	27 (17.1%)
6-OHDA lesioned (4 rats)	95 (83.3%)	19 (16.7%)

c

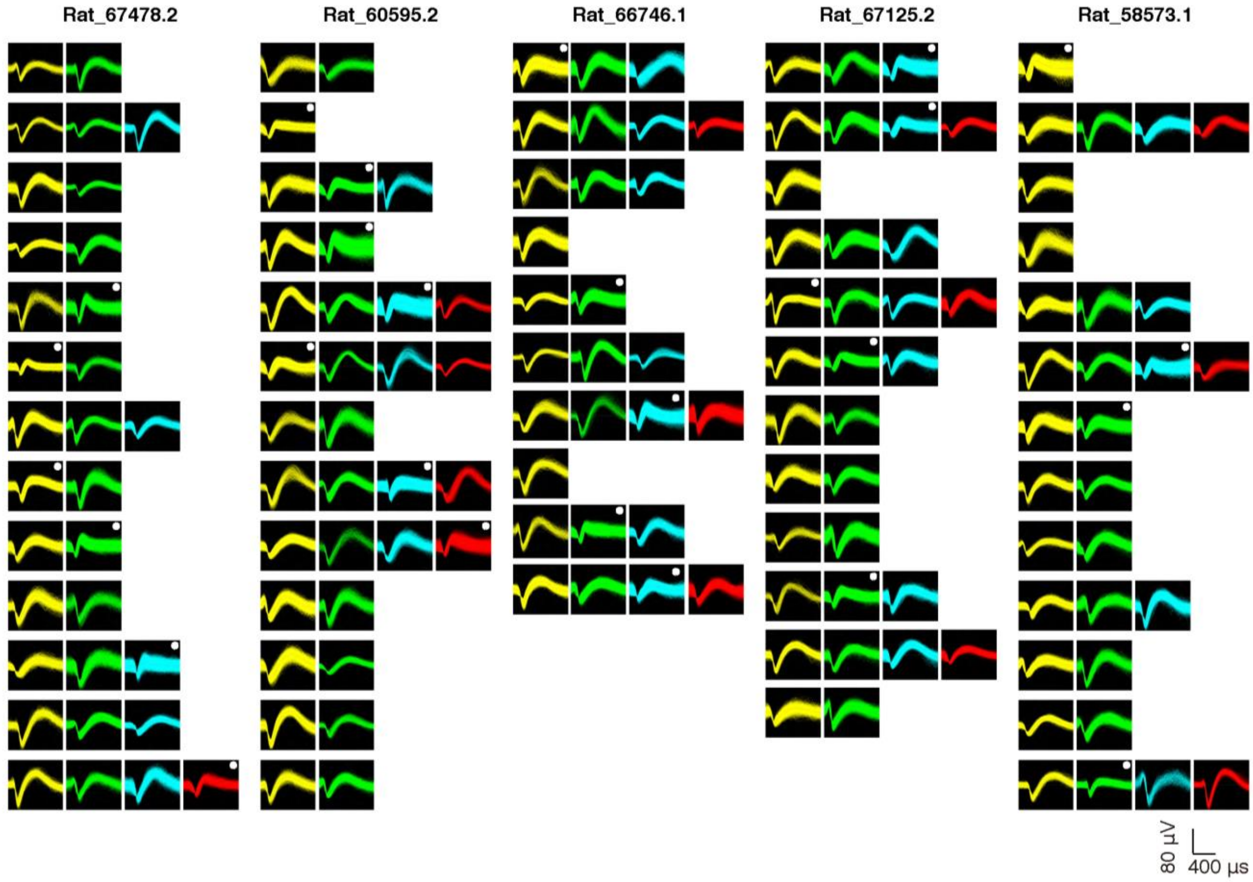

Supplementary Figure 3 Electrophysiological classification of putative pyramidal projection neurons (PNs) and interneurons (INs). (**a**) Neuron type identification. Top: spike waveforms for typical PN (red) and IN (blue) respectively. Fine lines: 50 raw traces; bold line: averaged trace. The time interval between two black dotted lines indicates the spike valley-to-peak amplitude width (‘t’, ms). ‘v’, ‘p’ mark spike valley and peak amplitude respectively. Bottom: Automatic clustering of putative PNs (*n* = 131, red) and INs (*n* = 27, blue) by K-means clustering algorithm based on three electrophysiological properties: spike valley-to-peak width (t, ms), valley-to-peak amplitude ratio (v/p) and mean firing rate (n = 5 rats). (**b**) Proportion of putative PNs and INs recorded from L5b of the M1 (n = 5 rats). (**c**) Superimposed spike waveform samples from 158 single units recorded in the L5b of M1 of 5 rats highlighted in **Supplementary Fig. 1d**. Single units from the same channel were arranged in the same row, denoted by different colors. Putative interneurons were indicated by white asterisk.

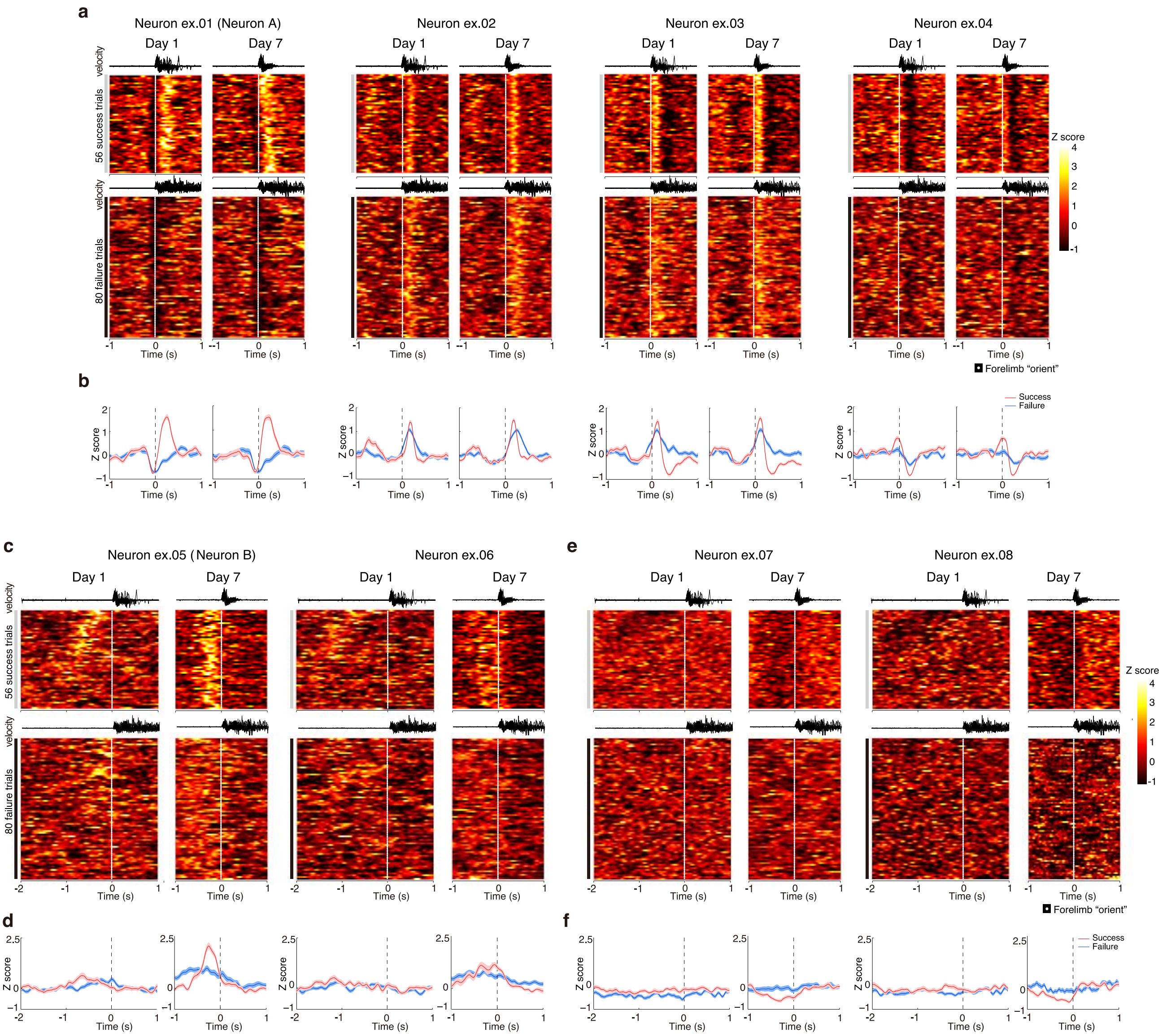

Supplementary Figure 4 Additional examples of L5b PN with consistent and training dependent firing patterns. (**a**) The peri-event time histograms of 4 typical PNs, whose behavior were characterized by the correlation of their firing patterns with the forelimb action. The corresponding forelimb velocity is shown overlaid on the top. Consecutive first reach success (grey bar) and failure (black bar) trials are stacked in rows from bottom to top and neural activities are normalized in Z sore. Time scale is restricted to 1s pre- and post- 'orient' (time '0', indicated by white dashed line). Neuron ex_01 was neuron A shown in the left panel of **Fig. 3a**. (**b**) Neural activity average of 4 L5b PNs shown in (**a**) during first reach success (red) and failure (blue) trials. **(c)** Two L5b PN examples showing training dependent increase of task correlated activity. Neuron ex_05 was neuron B shown in the middle panel of **Fig. 3a**. Consecutive first reach success (grey bar) and failure (black bar) trials are stacked in rows from bottom to top and neural activities are normalized in Z score. Time scale is restricted to 2s pre- and 1s post-forelimb 'orient' (time '0', indicated by dashed dot). (**d**) Neural activity average for each neuron shown in **(c)** during first reach success (red) and failure (blue) trials revealed a shortening of peak activity associated with a faster response time to reach the 'orient' position. (**e**) Two L5b PN examples showing training dependent emergence of decrease in activity, relative to forelimb 'orient' (dashed dot). (**f**) Neural activity average for each neuron shown in (**e**) during first reach success (red) and failure (blue) trials.

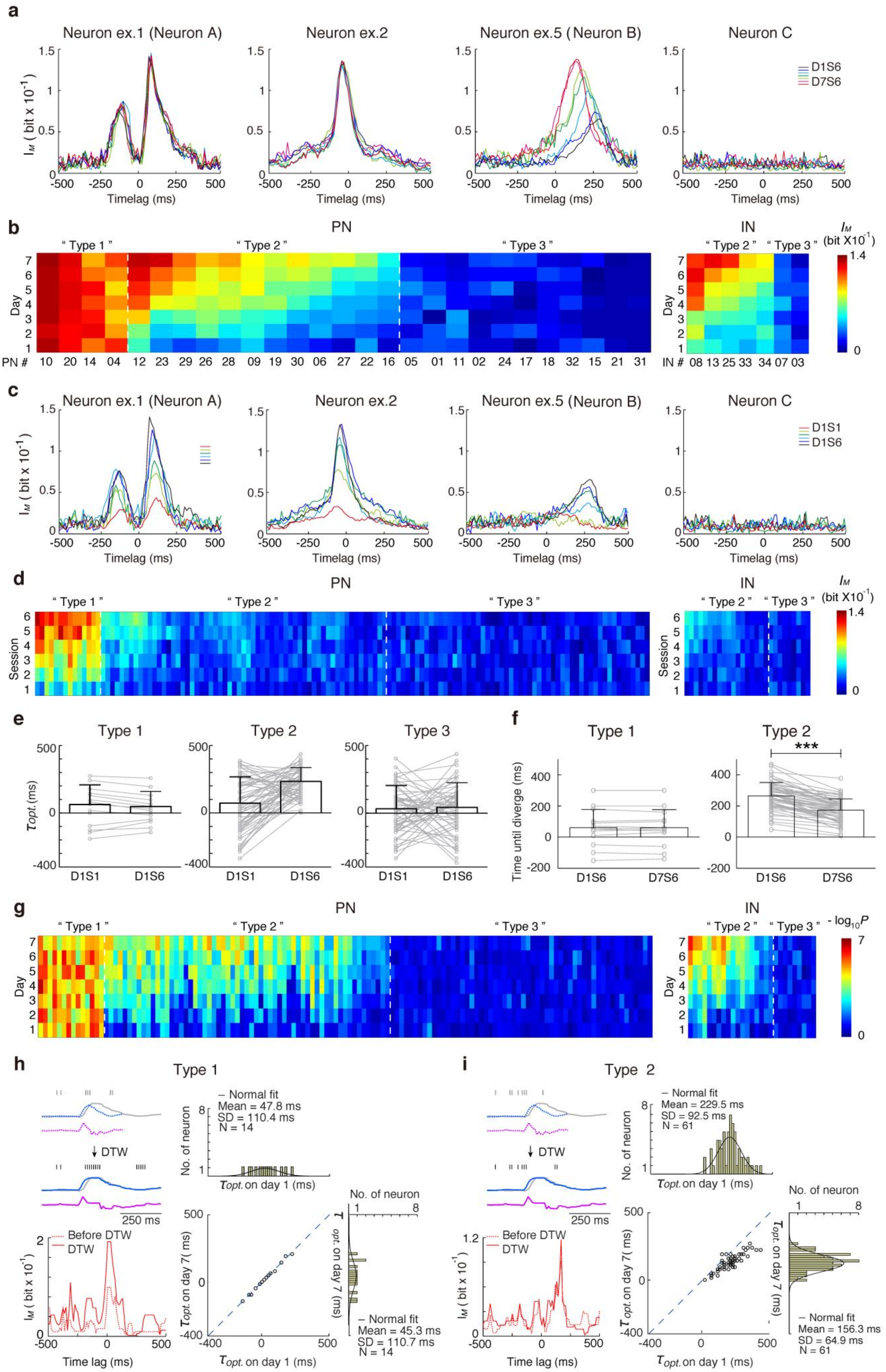
a
Neuron ex.1 (Neuron A)
Neuron ex.2
Neuron ex.5 (Neuron B)
Neuron C
D1S6
D7S6
I_M (bit x 10^-1)
Timelag (ms)
b
PN
IN
" Type 1 "
" Type 2 "
" Type 3 "
I_M (bit X10^-1)
Day
PN # 10 20 14 04 12 23 29 26 28 09 19 30 06 27 22 16 05 01 11 02 24 17 18 32 15 21 31
IN # 08 13 25 33 34 07 03
c
Neuron ex.1 (Neuron A)
Neuron ex.2
Neuron ex.5 (Neuron B)
Neuron C
D1S1
D1S6
I_M (bit x 10^-1)
Timelag (ms)
d
PN
IN
Session
e
Type 1
Type 2
Type 3
τopt. (ms)
D1S1
D1S6
f
Type 1
Type 2
Time until diverge (ms)
D1S6
D7S6
***
g
PN
IN
- log10P
Day
h
Type 1
DTW
250 ms
Before DTW
DTW
I_M (bit x 10^-1)
Time lag (ms)
– Normal fit
Mean = 47.8 ms
SD = 110.4 ms
N = 14
No. of neuron
τopt. on day 1 (ms)
τopt. on day 7(ms)
No. of neuron
τopt. on day 7 (ms)
– Normal fit
Mean = 45.3 ms
SD = 110.7 ms
N = 14
i
Type 2
DTW
250 ms
Before DTW
DTW
I_M (bit x 10^-1)
Time lag (ms)
– Normal fit
Mean = 229.5 ms
SD = 92.5 ms
N = 61
No. of neuron
τopt. on day 1 (ms)
τopt. on day 7(ms)
No. of neuron
τopt. on day 7 (ms)
– Normal fit
Mean = 156.3 ms
SD = 64.9 ms
N = 61

Supplementary Figure 5 Changes in the time-lagged mutual information (I_M) of single neuron firing activity and forelimb instantaneous velocity during training. (**a**) Four examples of L5b PNs showing changes in time-lagged mutual information (I_M) of firing activity and forelimb instantaneous velocity during the 7-day training period. The neural activity series is shifted bin by bin (bin size: 12.5 ms, from -500 to 500 ms) to assess the optimal time lag $\tau_{opt.}$. For positive lags, neural activity leads forelimb velocity. The four neurons correspond to the neurons shown in **Fig. 3a** (Neuron A, B and C) and one (Neuron ex.2) shown in **Supplementary Fig. 4a**. Neuron ex.1 exhibited one peak value of I_M with neuronal activity leading motor output and a smaller peak with motor output leading firing, whereas Neuron ex.2 only had one peak value of $I_M$with firing leading motor output. Neuron B's firing had a progressive increase in the peak value of I_M and shortening of $\tau_{opt.}$. (**b**) Hierarchical clustering of L5b 27 PNs' and 7 INs' peak value of I_M recorded from one typical animal, which are ordered according to the clustering sequence generated in **Fig. 3c,d**. Three main types of L5b PNs are determined and segregated by the white dashed lines. (**c**) The same four examples of L5b PNs as **(a),** showing the changes in time-lagged mutual information (I_M) of firing activity and forelimb instantaneous velocity during 6 training sessions on day 1. **(d)** Hierarchical clustering of L5b 27 PNs' and 7 INs' peak value of I_M during 6 sessions on day 1, which are ordered according to the clustering sequence generated in **Fig. 3c,d**. **(e)** Training dependent changes of $\tau_{opt.}$ from first to last session on day 1. **(f)** Training dependent changes in time until divergence (TUD, referred to the time of forelimb 'orient') in the firing of type 1 and type 2 PNs. After 7 days of training, significant shortening of TUD was found only among type 2 PNs (TUD in day 1 session 6: 265.1 ± 85.6 ms, day1 session 6: 173.1 ± 72.0 ms, mean ± s.d., paired *t*-test, $P < 10^{-5}$). (**g**) Statistical significance of single neuron I_M of all 131 PNs and 27 INs from 5 rats (*P*-values plotted in -log-10 scale) is estimated by a bootstrap resampling procedure. The neurons are ordered according to the clustering sequence generated in **Fig. 3c,d**. The white lines segregate the three types of PNs. (**h**) and **(i)** Quantification of the relationship between $\tau_{opt.}$ and reach duration. Top left: Neural firing activities (dashed black), forelimb movement displacement (dashed blue) and corresponding instantaneous velocity (dashed pink) are extracted from each reaching attempts (-250 ms to 250 ms from 'orient'). DTW is performed to find the optimal warping for each reach trajectory referred to the reference expert trajectory (grey, the average of 50 randomly chosen trajectories in day 7 session 6). Individual time series of neural spikes (black), forelimb movement displacement (blue) and corresponding instantaneous velocity (pink) were stretched applying the optimal path to control the reach action variability. Bottom left: time-lagged mutual information between single neural activity and velocity is recalculated. Right: Summary of training dependent changes of $\tau_{opt.}$for type 1 and type 2 neurons after DTW. Type 2 neurons maintain the systematically shortened $\tau_{opt.}$ with training.

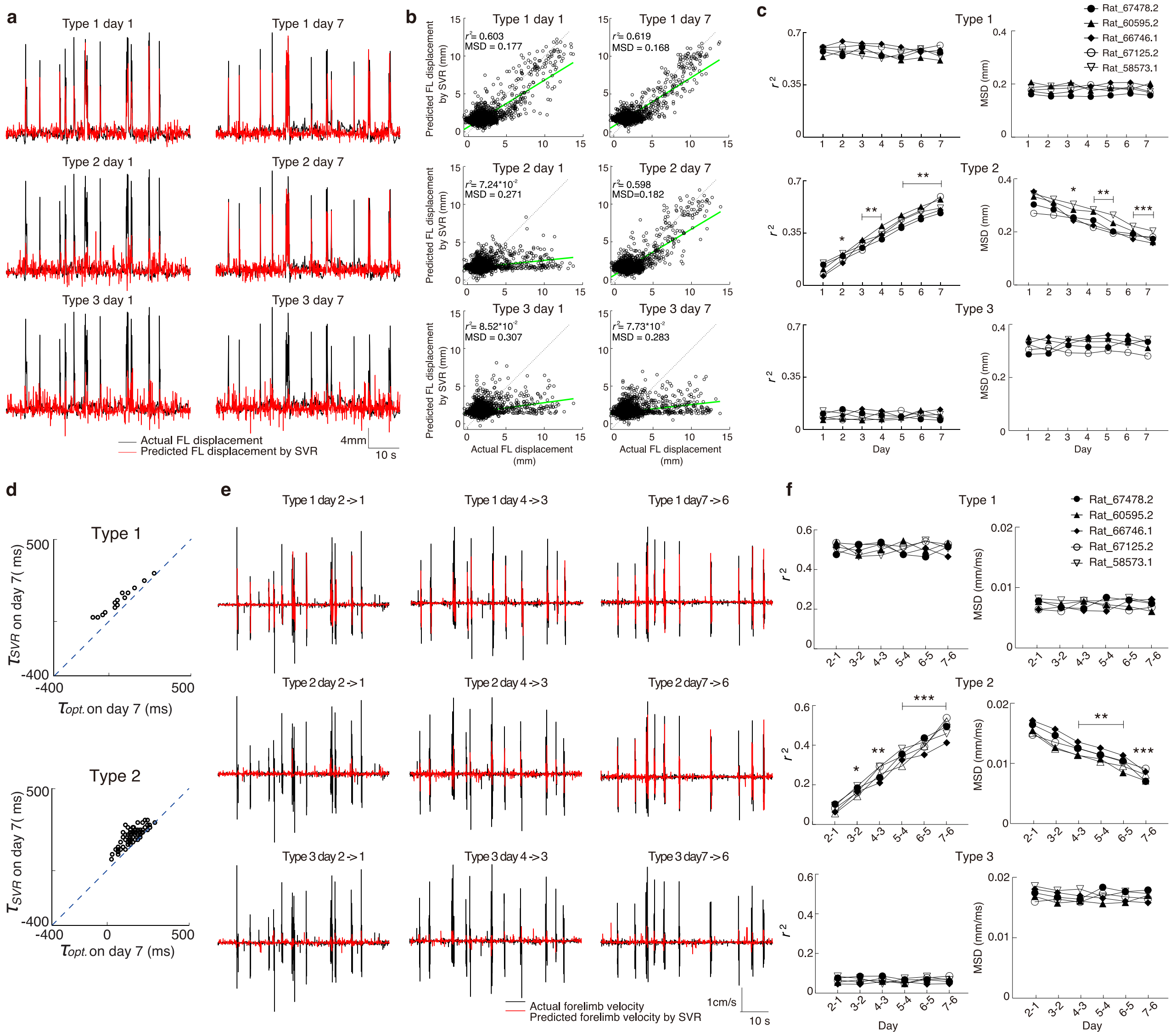

Supplementary Figure 6 Population decoding of forelimb displacement by three types of L5b PNs. (**a**) Actual forelimb movement displacement (black) and predicted forelimb displacement (red) by SVR model (see Methods) based on three types of PNs' neural population activities (type 1 neuron: n = 4; type 2 neuron: n = 12; type 3 neuron: n = 11, from an representative animal). (**b**) The least squares regression between actual forelimb displacement and the SVR model predicted forelimb displacement by three types of PNs' neural population activities shown in **(a)**. The Pearson's correlation coefficients r^2 and MSD for each regression are shown. (**c**) Prediction accuracy is evaluated by r^2 and MSD. **(d)** Distribution of single neuron $\tau_{opt.}$ for mutual information and the τ_{SVR} for maximal prediction accuracy. (**e**) Representative traces of actual forelimb instantaneous velocity (black) and the SVR model predicted forelimb velocity (red). The model is trained with data from each day's last session and applied to the previous day. (**f**) Summarized results of cross-day SVR prediction accuracy. Top panels, mean ± s.e.m. of type 1 neuron previous-day prediction correlation coefficient r^2: day-2 predicted day-1: r^2=0.512±0.012, P=0.948, compared to day-1 same-day prediction; day-7 predicted day-6: r^2=0.510±0.015, P=0.706, compared to day-6 same-day prediction, paired-t test; mean ± s.e.m. of MSD predicted by previous-day velocity: day-2 predicted day-1: 0.0073±0.00037, P=0.617, compared to day-1 same-day prediction; day-7 predicted day-6: 0.0071±0.00034, P=0.769, compared to day-6 same-day prediction; paired-t test. Middle panels: mean ± s.e.m. of type 2 neuron previous-day prediction correlation coefficient day-2 predicted day-1: r^2=0.081±0.0092, P=0.129, compared to day-1 same-day prediction; day-7 predicted day-6: r^2=0.486±0.022, P=0.026, compared to day-6 same-day prediction, paired-t test; mean ± s.e.m. of MSD predicted by previous-day velocity: day-2 predicted day-1: 0.0158±0.00044, P=0.034, compared to day-1

same-day prediction; day-7 predicted day-6: 0.0079±0.00041, compared to day-6 same-day prediction, *P*=0.067, paired-*t* test. Bottom panels: mean ± s.e.m. of type 3 neuron previous-day prediction correlation coefficient and SMD.

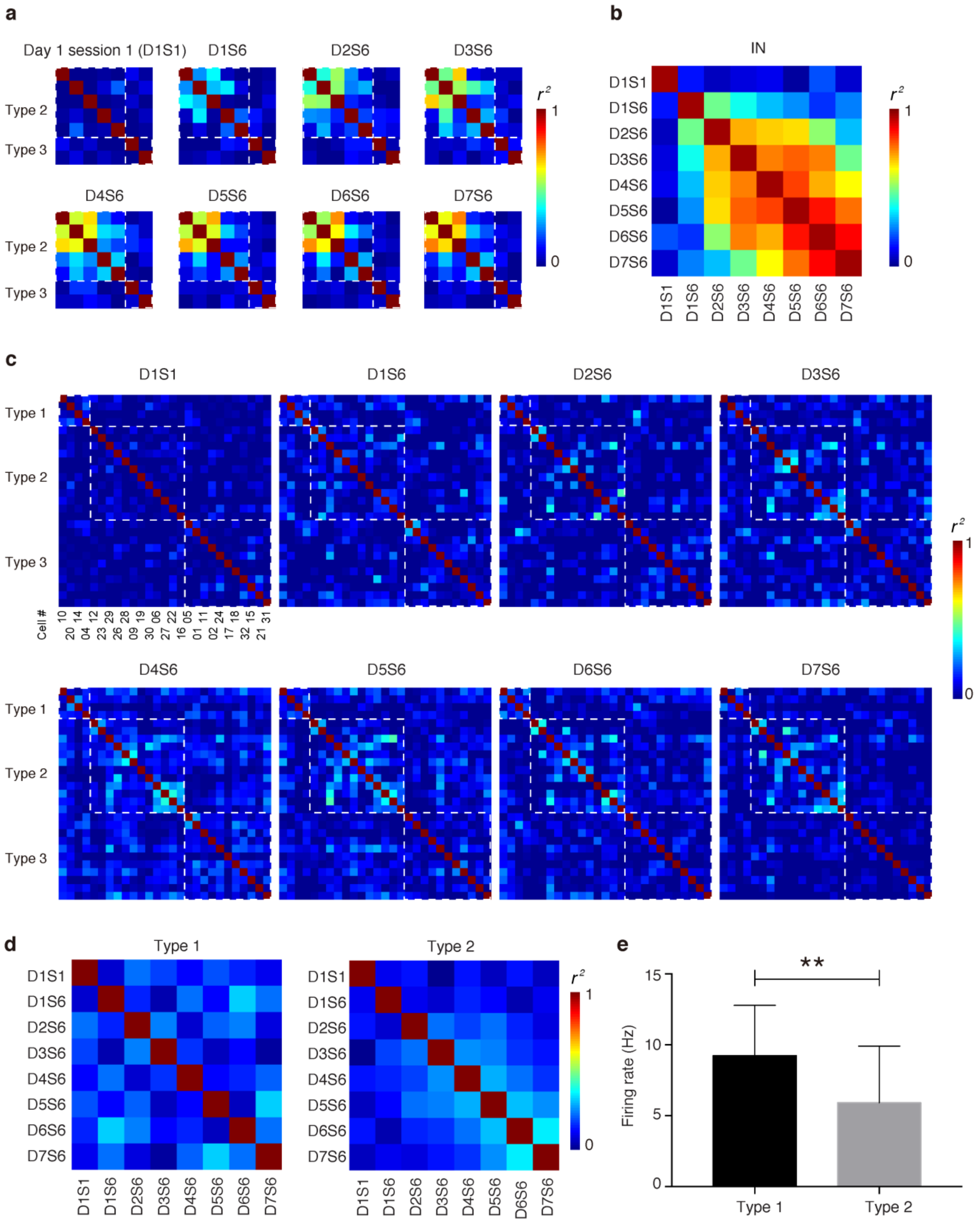

Supplementary Figure 7 Temporal evolution of correlation structure among L5b neurons. (a) Pairwise correlation matrices among 7 L5b INs across 7 training days, recorded from one representative rat during variation controlled first reach success attempts (see Methods). **(b)** Summary of the preserved overall similarity of cross-day correlation matrices among INs. Each color-coded element represents the averaged similarity index of cross-day correlation matrices from 5 rats. (**c**) Spontaneous firing rate for type 1 and type 2 L5b PNs. **(d)** Pairwise correlation matrices among the 27 L5b PNs shown in **Fig. 5a**, computed from inter-session spontaneous firing. **(e)** Pooled overall similarity of the cross-day correlation matrices among type1 and type 2 PNs during spontaneous firing when animals were not executing the task.

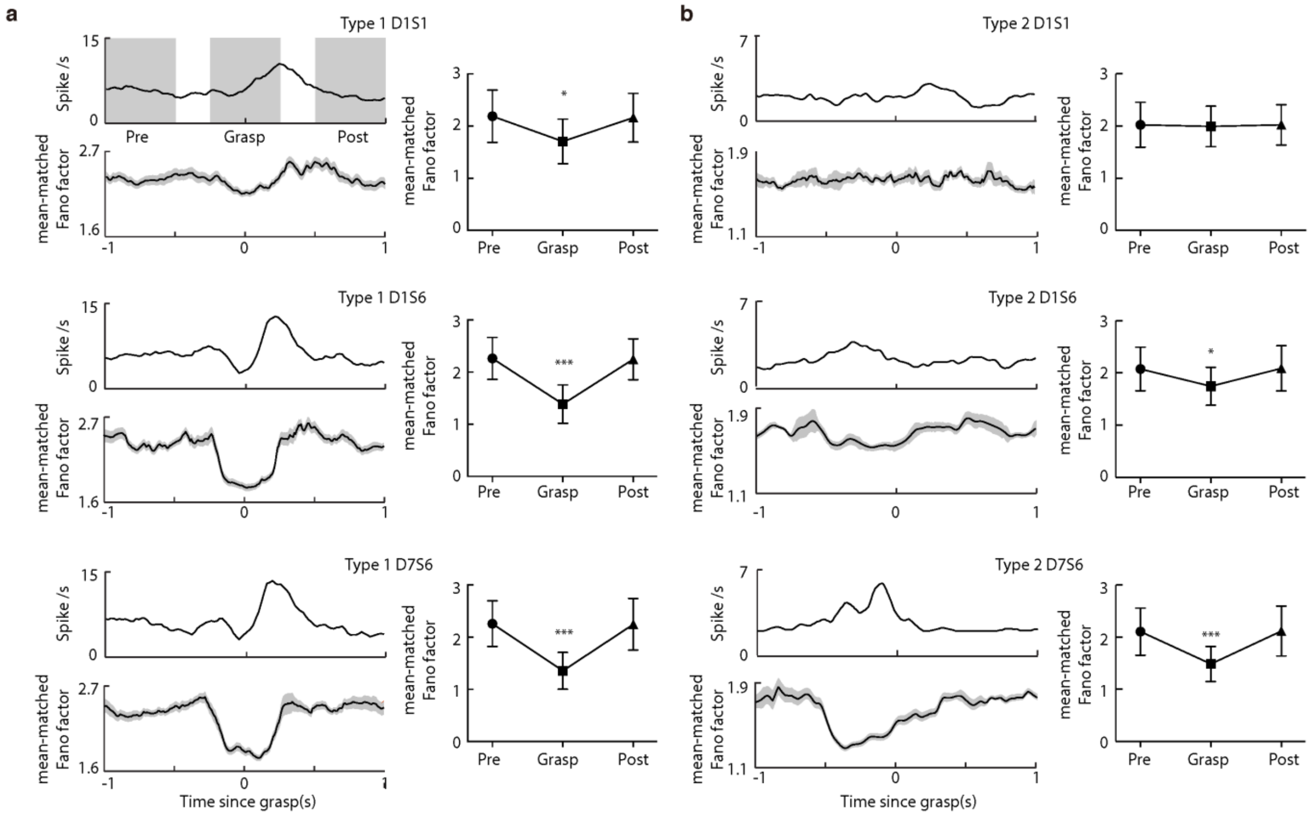

Supplementary Figure 8 Evaluation of neural firing variation in different motor training days. Mean-matched Fano Factor (FF) for type 1 (**a**) and type 2 (**b**) neuron during first reach success trials, which is calculated by dividing the variance by the mean in a 50-ms sliding windows moving in 10-ms increments (see Methods). Top left for each panel: the mean firing rate during 1s pre- and post- first reach success trials, which is aligned to grasp time (time ‘0’). Shaded areas mark three epochs for accessing firing variation before (-1 to -0.5), during (-0.25to 0.25 s) and after (0.5 to 1 s) grasping. Bottom left for each panel: the correspondent mean matched FF (black with flanking 95% confidence intervals). Right: Statistical significance of the mean matched FF from baseline at 3 different behavior episodes defined by: pre-, during and post-grasp.

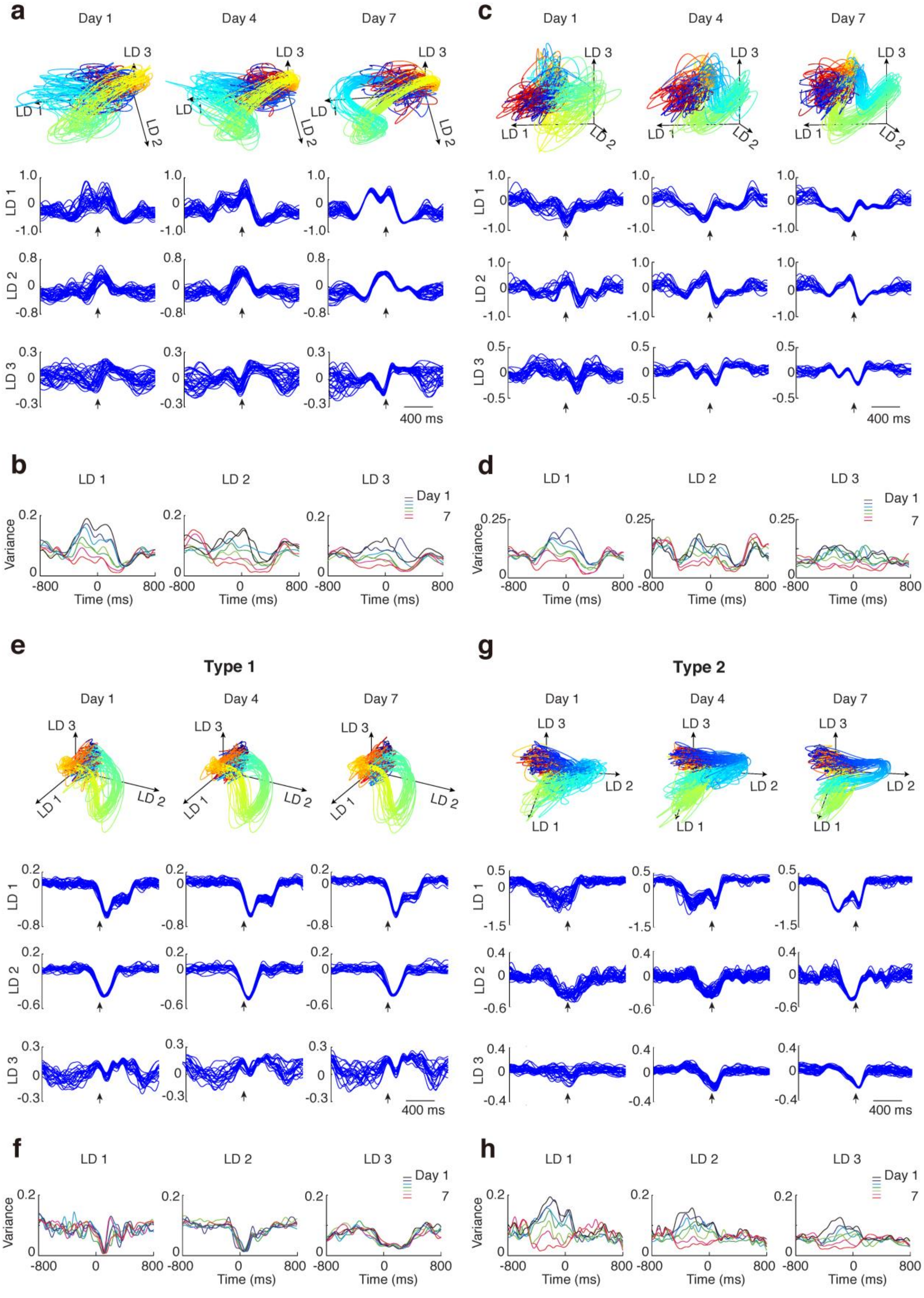

Supplementary Figure 9 Additional examples showing training dependent emergence of uniform single-trial based neural trajectories extracted by GPFA. For each day's training, the cross-trial variability of forepaw movement is controlled for by sub-selecting top 30 first reach success trials whose trajectory deviation is within mean ± s.d. cumulative Euclidean distance to reference expert trajectory **(a)-(d)**, Single-trial neural trajectories extracted from neural population activities of another two animals. **(e)** and **(f)** Neural trajectories of type 1 neurons**. (g)** and **(h),** Neural trajectories of type 2 neurons. Only type 2 neurons exhibited reduced neural trajectory variability in latent dimensions over training.

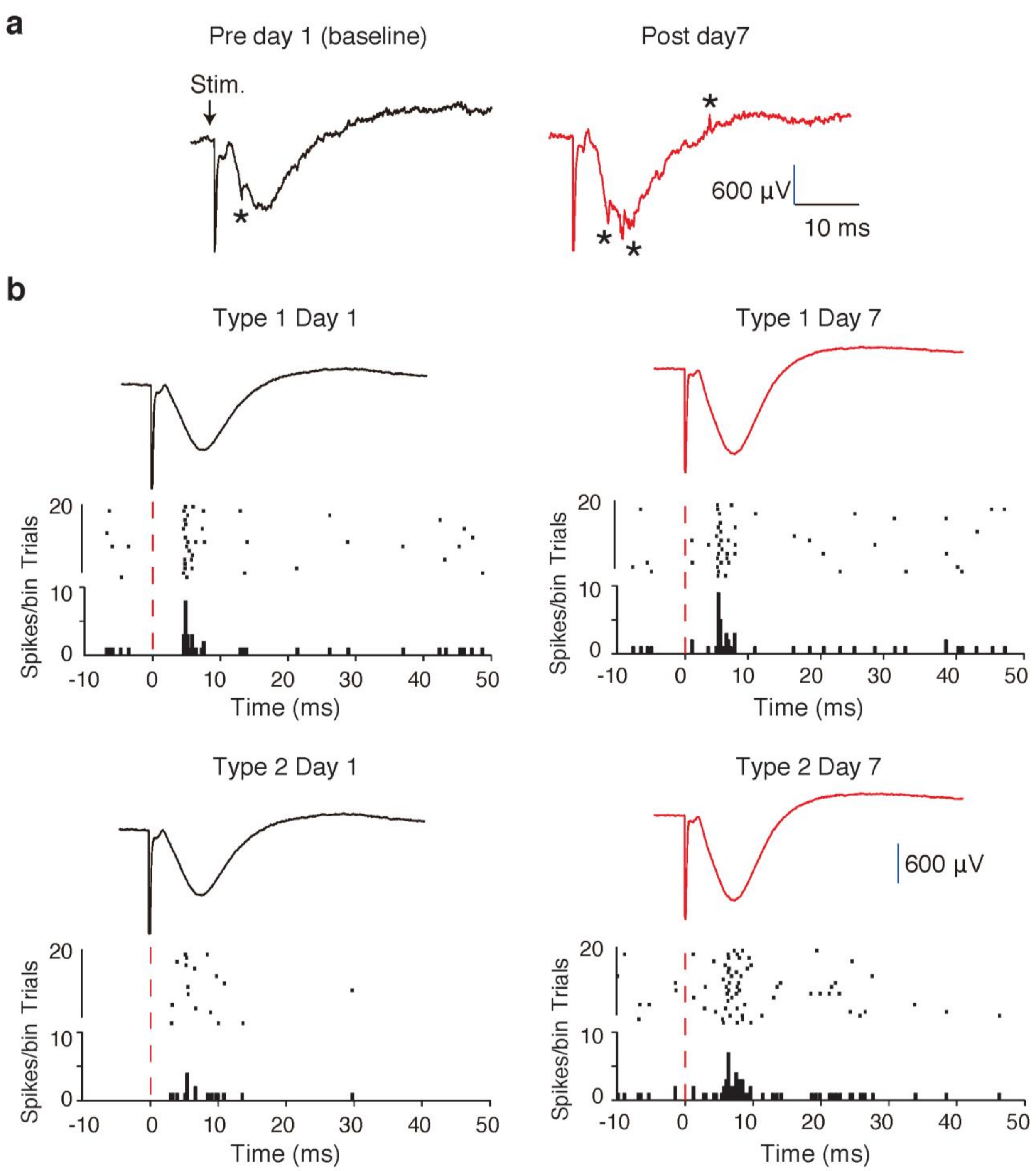

Supplementary Figure 10 LFPs and single neural firing on different training days. (a) Typical stimulation evoked raw traces. Unit spikes (indicated by '*') and band-pass filtered LFPs (0-5 kHz) are recorded simultaneously from the same electrode. **(b)** Raster plots and peri-stimulus time histograms of single-unit responses and peri-stimulus average of LFPs evoked by 20 inter-trial stimulation. Increased single-unit activities accompanied the negative deflections of LFPs. Only type 2 neurons exhibited enhanced phase locking phenomenon after 7 days' training.

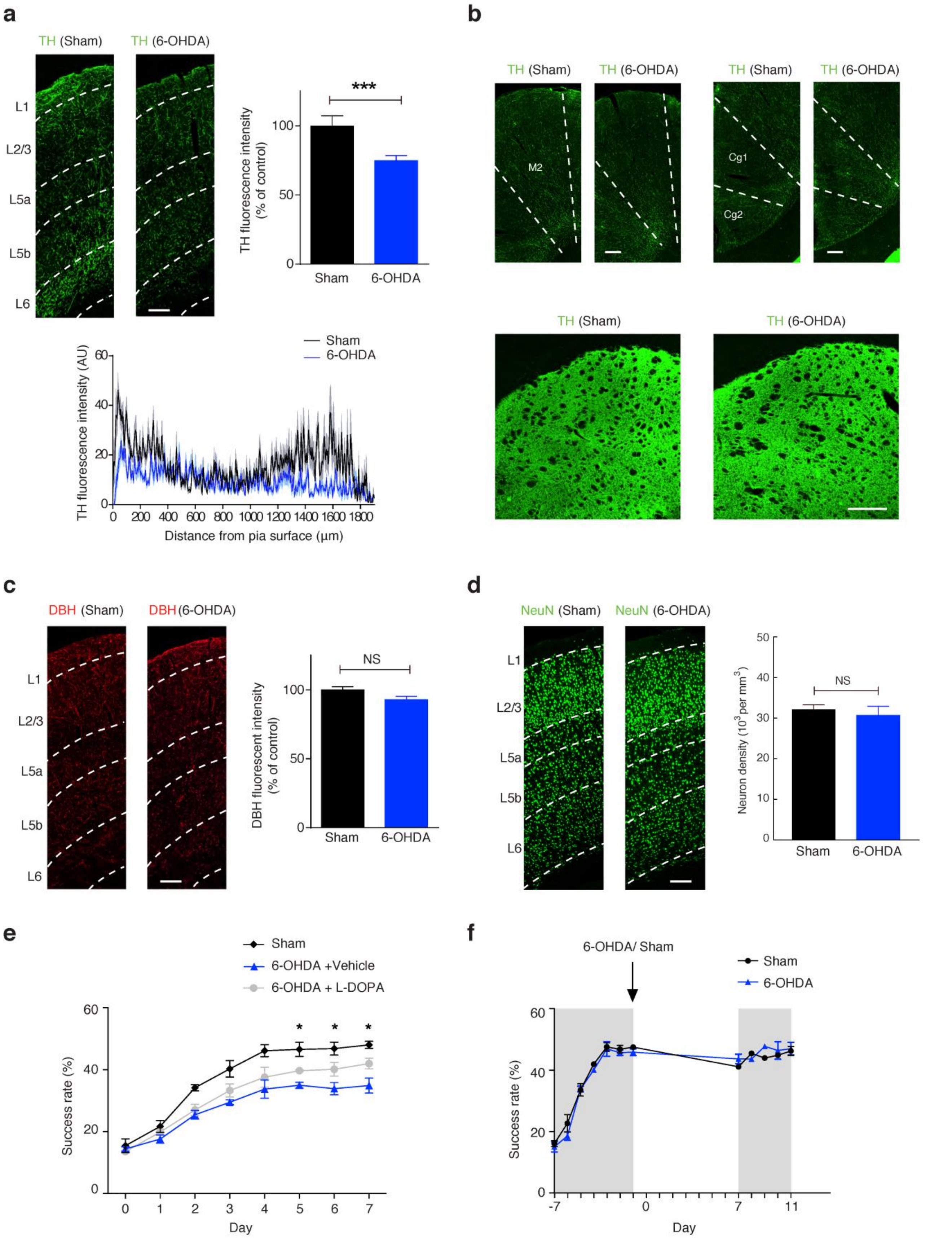

Supplementary Figure 11 Histological verification of specificity of dopamine depletion paradigm in M1. (**a**) Top, representative tyrosine hydroxylase (TH) immunofluorescence staining of cortical catecholaminergic terminals in vehicle-injected (sham, left) and 6-OHDA lesioned hemisphere (6-OHDA, right). A clear and significant reduction of TH-positive terminals from L1 to L5 of the M1 was found

(mean±s.e.m., one-way ANOVA, $P < 0.001$, sham: 11 slices from 3 rats; 6-OHDA: 13 slices from 3 rats). Bottom, quantification of TH-positive optical density from pia to L6 of M1, comparing sham (18 slices from 3 rats) and 6-OHDA injected hemisphere (24 slices from 3 rats). The data are averaged from 25 slices of 3 rats. AU: arbitrary unit. Scale bar = 200 μm. (**b**) TH-terminal reaching secondary motor cortex (M2), cingulate cortex, area 1(Cg1), cingulate cortex, area 2 (Cg2) and the dorsal striatum were not affected by 6-OHDA local injection restricted to M1. Scale bar: 200 μm (top) or 500 μm (bottom). (**c**) Dopamine-beta-hydroxylase (DBH) staining showed that noradrenergic terminals were spared either at sham and 6-OHDA lesioned site. Scale bar: 200 μm. (**d**) NeuN-staining staining illustrated normal neuronal density in L5b after cortical dopamine denervation, confirmed by statistical analysis of neuron number per mm^3. (mean ± s.e.m., one-way ANOVA, $P = 0.376$, sham: 10 slices from 3 rats; 6-OHDA: 17 slices from 4 rats). The NeuN density in L5b M1 was comparable between sham operated and 6-OHDA lesioned cortex. Scale bar = 200 μm. (**e**) Administration of L-DOPA during motor learning in 6-OHDA treated rats could partially restore degraded motor learning. Mean ± s.d. of first success rate on day-7: 6-OHDA+vehicle: 34.8±1.6%, n=3 rats, 6-OHDA+L-DOPA: 41.9±2.9%, n=3 rats, P=0.035; sham: 48.1±2.2%, n=4 rats, P=0.021; all compared to 6-OHDA+vehicle group, Kruskal-Wallis H test. (**f**) In rats in which 6-OHDA was administered after 7 days of training, no difference in performance was detected compared with the sham control.
